# Towards a low CO_2_ emission building material employing bacterial metabolism (2/2): Prospects for global warming potential reduction in the concrete industry

**DOI:** 10.1371/journal.pone.0208643

**Published:** 2019-04-16

**Authors:** Anders Myhr, Frida Røyne, Andreas S. Brandtsegg, Catho Bjerkseter, Harald Throne-Holst, Anita Borch, Alexander Wentzel, Anja Røyne

**Affiliations:** 1 Pure Logic AS, Oslo, Norway; 2 RISE Research Institutes of Sweden, Göteborg, Sweden; 3 Consumption Research Norway, Oslo Metropolitan University, Oslo, Norway; 4 Department of Biotechnology and Nanomedicine, SINTEF Industry, Trondheim, Norway; 5 Department of Physics, University of Oslo, Oslo, Norway; Karl-Franzens-Universitat Graz, AUSTRIA

## Abstract

The production of concrete is one of the most significant contributors to global greenhouse gas emissions. This work focuses on bio-cementation-based products and their potential to reduce global warming potential (GWP). In particular, we address a proposed bio-cementation method employing bacterial metabolism in a two-step process of limestone dissolution and recrystallisation (BioZEment). A scenario-based techno-economic analysis (TEA) is combined with a life cycle assessment (LCA), a market model and a literature review of consumers’ willingness to pay, to compute the expected reduction of global GWP. Based on the LCA, the GWP of 1 ton of BioZEment is found to be 70–83% lower than conventional concrete. In the TEA, three scenarios are investigated: brick, precast and onsite production. The results indicate that brick production may be the easiest way to implement the products, but that due to high cost, the impact on global GWP will be marginal. For precast production the expected 10% higher material cost of BioZEment only produces a marginal increase in total cost. Thus, precast production has the potential to reduce global GWP from concrete production by 0–20%. Significant technological hurdles remain before BioZEment-based products can be used in onsite construction scenarios, but in this scenario, the potential GWP reduction ranges from 1 to 26%. While the potential to reduce global GWP is substantial, significant efforts need to be made both in regard to public acceptance and production methods for this potential to be unlocked.

## Introduction

### Background

The construction sector is a major contributor to global warming, responsible for around 18% of global greenhouse gas emissions [1]. Concrete is the most extensively used construction material, with a global production of around 10 km^3^ of concrete every year [2]. The most common applications of concrete are in the form of bricks, as factory-casted structural elements (precast), or as structures casted in forms directly on the construction site.

Concrete is made of water, aggregate (coarse and fine particles such as gravel and sand), and cement, the latter which acts as a binder between the particles in the aggregate. In most applications, concrete is reinforced with steel to enhance its mechanical properties. Steel reinforcement is typically preferred over alternatives such as plastic or glass fibres due to its relatively low cost and high strength. The pH of concrete is typically around 12, which leads to the formation of an impermeable oxide layer on the steel surface. This acts as a corrosion barrier, making the composite more durable, with an achievable service life of over 100 years.

A main ingredient in most common cements is lime (CaO), which is produced via the high-temperature calcination of crushed limestone (CaCO_3_). The fossil CO_2_ released from limestone during calcination, and from the fossil fuels used to provide heat for the calcination process, currently account for more than 5% of global anthropogenic CO_2_ emissions. Global cement production has increased from nearly 1.6 Gton in 2000 to 4.1 Gton in 2016 [3]. The large climate impact, combined with the increasing demand for concrete, make it hugely important to develop more sustainable solutions for the construction industry.

This study investigates the potential of new types of biotechnology-based concrete materials as sustainable alternatives to traditional concrete with reduced global warming potential (GWP). We propose a concept named BioZEment, with the capital letters ‘ZE’ as an acronym for 'Zero Emission' to underscore our efforts of producing a low CO_2_ emitting cement. BioZEment has undergone an initial technical feasibility and development phase as detailed in [4]. However, in order to have global impact, BioZEment-based materials need to prove more than just technical feasibility. Global impact relies on large-scale adoption, which requires the product to be perceived as environmentally sustainable, economically desirable, socially desirable and acceptable, technically feasible, and safe.

To identify the potential of these products, a techno-economic uncertainty analysis is used to predict possible outcome distributions under various product scenarios for GWP reduction. The analysis relies on the inputs and assumptions from the technical development of the BioZEment process [4], a life cycle assessment (LCA), cost analyses, customer preferences, and market modelling in order to predict a potential market share and thereby possible reductions in GWP.

### Bio-cementation

The use of biotechnology in the manufacture of construction materials is regarded as a promising method for reducing the environmental impacts of the construction industry because these materials can be made with renewable energy sources and low temperature requirements. Most recent approaches for using biotechnology in the construction industry are based on 'microbial-induced carbonate precipitation' (MICP) [5, 6]. Several bacterial species, including various *Bacillus spp*. and the most prominent MICP species *Sporosarcina pasteurii*, can induce the precipitation of calcium carbonate minerals in the presence of carbonate and calcium ions through metabolic processes that increase the pH of their surroundings [7], for example, by employing the enzyme urease (urea amidohydrolase; EC 3.5.1.5). Urease catalyses the hydrolysis of urea, and the pH increase and generation of carbonate ions that results from this reaction can lead to the precipitation of carbonate minerals that act as binders between aggregate grains. Research on MICP is ongoing for applications including stone conservation [8], crack remediation in concrete [9, 10], cement mortar [2, 11], soil stabilisation [12], self-healing concrete [13], contaminant immobilisation [14], and the construction of subsurface barriers against pollutant spread or reservoir leakage [15]. However, despite significant efforts, the use of bio-cementation has yet to be adopted in large-scale applications that have a significant impact on global CO_2_ emissions. The challenges that must be managed before adoption within to large-scale applications include sufficient mechanical strength, fast production times, controlled shrinkage and creep, and a cost-efficient and environmentally sustainable large-scale production process. It is also important to note that the pH of carbonate-based bio-concrete materials is not sufficiently high to form the corrosion barrier that is necessary to protect the steel in reinforced concrete structures. This places additional limitations on the applications where bio-cement can be expected to be used as a substitution for conventional cements.

### The BioZEment approach

Bio-cement made by MICP requires suitable bacteria, nutrients, urea, and a calcium source. Finding a low-cost calcium source that does not adversely affect material properties has been identified as one of the key challenges for the upscaling of bio-cementation to industrial scales [5]. In the BioZEment concept, dedicated non-pathogenic bacteria strains, such as those belonging to the genus *Bacillus*, are used to produce acids (predominantly lactic and acetic acid) that lower the pH and induce calcium carbonate dissolution, leading to the liberation of free calcium ions from powdered limestone (composed primarily of calcium carbonate). The low cost and high global abundance of limestone makes it a favourable raw material for concrete production, and the ability to use it directly as a major ingredient in concrete without having to first calcinate it at high temperature would significantly reduce climate gas emissions [5].

In the BioZEment process, bacteria are used to transform calcium carbonate from crushed material into a binder in a low temperature process. We have isolated an alkali-tolerant, acid-producing bacterial strain, denoted as AP-004 and based on 16S rRNA gene amplicon sequencing closely related to the *Bacillus safensis* strains FO-35b and NBRC 100820, from a soil sample taken close to an open chalk quarry in central Norway that we have found to be suitable for this dissolution process. The strain produces predominantly lactic acid and acetic in the pH range of 9.5 to 6.0, as detailed in [4]. In the subsequent step, urea is added together with the urease-producing strain DSM33 of the bacterium *Sporosarcina pasteurii*, *obtained from the ‘Deutsche Sammlung von Mikroorganismen und Zellkulturen’ (DSMZ)* and commonly used in MICP applications (see [4] for details). The *S*. *pasteurii*-derived urease-catalysed hydrolysis of urea induces an increase in pH, which leads to the precipitation of the previously microbially dissolved calcium carbonate. The precipitated material acts as a binder between grains of sand and other aggregate materials, including residual non-dissolved calcium carbonate grains. The process and feasibility of this two-stage process of dissolution and re-precipitation is further discussed and documented in [4]. At the current stage of development, the formation of a solid material relies on multiple injections of the dissolved limestone solution into the aggregate materials, something that is possible in the production of bricks and precast concrete structures. However, we envision that the need for multiple injections can be eliminated through further developments of the process, which could ultimately allow for the onsite casting of structures. We have therefore included this process in this study.

### Potential for introducing BioZEment-based products

A product with low emissions is likely to have an impact on global emissions if it is significantly cheaper and equal to or more practical than the alternatives, thus enabling large-scale application. Thus, to unlock the potential emission savings, the product will have to outperform its competitors in more aspects than merely environmental benefit. There are several ways in which a new product can be successful in an existing market, such as being perceived as better or cheaper. A new product can fulfil an existing or emerging consumer demand, and in this aspect, BioZEment-based materials may be considered new and interesting alternatives in the recent surge for more sustainable building materials. The use of harmless, non-pathogenic, non-genetically modified organisms (GMO) bacterial strains in the BioZEment process producing easily degradable acids and enzymes and no other potentially environmentally hazardous metabolites are important features for the perception of BioZEment-based materials as a beneficial alternative.

In the introduction phase of a new product, the production volume is often low, making it challenging to compete with existing products in terms of cost. To some extent, this can be compensated by a positive consumer perception towards a new product with new features, currently in particular with a ‘green’ profile. On the other hand, public perceptions of a product being potentially harmful to human health or the environment (as evident from the wide-spread debates on GMOs) may have an adverse effect on demand and sales.

Concrete is, in general, a low-cost material. Sand, gravel, and water are widely available at or close to most production sites, and the experience with regard to tuning and optimisation with various additives is the result of literally centuries of experimentation. Cement is the most expensive component in a concrete mix, both in terms of monetary cost and environmental footprint. The most commonly used cement, Portland cement, has been used since the 1840s [16], and the general recognition of and knowledge on this material is high. The material properties of concrete may be altered and optimised in several dimensions, such as cost, properties in the liquid phase, and properties in the solid state. The low cost and flexible properties of concrete may be the main reason why there has not been a more sustainable development in concrete production [17] over the past decades, despite of the increased focus and awareness of the environmental footprint of concrete production.

Ready-mixed concretes account for about 50% of the cement used for concrete products although large variations exist at the local scale as it is used for both onsite casting and precast products [17]. Onsite concrete construction is applied in civil structures, such as dams and buildings, and in structural elements, such as bottom-plates, floors, and walls. These are typically ‘simple’ elements that do not require high mechanical strength, and they require only limited reinforcement, although structures without any form of reinforcement are rare. The incompatibility with steel reinforcements could be a significant setback for calcium carbonate cement-based concrete materials. However, there are a variety of alternative fibre reinforcements commercially available. Elements that are designed to take higher loads, such as beams and long floor spans, are often precast offsite and reinforced with post-tensioned steel. Post-tensioning reinforcement systems are commonly installed in plastic ducts, separating the steel from the concrete, and would therefore be compatible with the chemical environment of calcium carbonate cements. These types of structures require higher mechanical strength, but they are typically built in factory-like environments, where the casting process can be closely monitored and controlled. Concrete bricks and tiles are typically also manufactured offsite in specialised factories and account for about 20% of the global concrete use [17]. Concrete bricks are not very durable and typically have lower mechanical strength than clay bricks but are widely used for the construction of plain structures and facades. The commonly desired qualities and attributes for each of the main cement applications are summarised in Table 1.

**Table 1:** Summary of important properties and aspects for various cement applications.

Application	Mechanical strength	Reinforced	Production & application environment
**Onsite concrete**	Low to medium	Most	Onsite
**Precast concrete**	High	Yes	Automated factory
**Brick & masonry**	Low to medium	No	Semi-automated factory

### Aim and approach

The aim of the present study was to investigate whether BioZEment products have the potential to make a significant impact on global GWP reduction. Most studies addressing alternative cements have not taken the cement’s potential market uptake into consideration. We argue that in order to reduce the risk of market failure, the potential market uptake has to be examined and considered from the very beginning of product development. Therefore, we applied a holistic approach to explore the potential market uptake of a few selected BioZEment-based products. This is performed in a techno-economic analysis (TEA) that receives input from the following four models: 1) LCA (environmentally responsible), 2) cost analysis (economically robust), 3) consumer studies (socially acceptable and desirable), and 4) market penetration model (adaption of the product).

TEAs are often used to justify technological development and projects and are applied in a wide range of fields, from biotechnology [18] to engineering [19]. It is commonly acknowledged that for a product to succeed, it needs to be feasible and competitive with respect to technical and economic aspects. In BioZEment’s development, engineering, environmental, economic, social science, and biotechnology disciplines are combined to produce a joint result that can illustrate the economic impact of decisions made during product development. However, such analyses usually rely on deterministic inputs and assumptions that do not consider the inherent uncertainty in the input factors. This poses a challenge for novel concepts in an early development phase, where several aspects of the production are subject to a significant amount of uncertainty. Still, important decisions, with impact on the future product, need to be made nonetheless in this early phase.

A TEA that properly takes all the relevant uncertainties into account can act as a powerful tool in determining the possible effect of early-stage decisions on the impact potential of the final product. In this study, we have considered the uncertainty in all relevant input elements of the analysis through the use of distributions for all inputs and assumptions in combination with a Monte Carlo approach.

Based on our current data, BioZEment-based products are assumed to be technically feasible, as discussed in [4]. Therefore, this study investigated the sub-goals of identifying 1) the total potential reduction of GWP under a given set of scenarios, 2) driving uncertainties limiting the potential to reduce GWP, and 3) possible strategies to enhance the potential GWP reduction.

Three scenarios for BioZEment implementation have been evaluated, including 1) brick production, 2) prefabrication production, and 3) onsite manufacturing. For each of the scenarios, the estimated unit cost is compared to existing substitutes. A probable market share is then calculated based on an estimated production volume for the given product segment and a sales price that is affected by assumptions of public acceptance. Public acceptance is an assumed important factor that is operationalised as an average customer’s willingness to pay a higher price for a ‘green’ product and the customer’s reluctance to buy something that can be perceived as potentially harmful to the environment or human health. The predicted market share is then used to compute the possible GWP reduction, which is taken as the difference in GWP between the products that are displaced and the corresponding BioZEment-based products.

## Methods

A TEA is commonly used to obtain a holistic overview of the technical parameters or boundary conditions that have the most influence on the performance of a project or product. TEAs are particularly valuable for complex projects or processes, where several different disciplines intersect or where there are clear or unclear dependencies between different assumptions and/or boundary conditions. To compare and investigate the implication of the interaction between the disciplines, assumptions, and boundary conditions on equal terms, their influences are estimated in terms of time and cost. Dependencies can be solved either by an integrated analysis where all parameters are dynamically interchanged between disciplines and assumptions or by the use of correlation matrices. In economic terms, this is similar to co-integration, but we prefer causal relations in order to better capture complex non-linear dependencies.

To predict GWP reduction, the TEA integrates results from the technical development, LCA studies, public acceptance considerations, and cost analysis. We used a semi-integrated approach, where the most relevant disciplines were integrated, but not all parameters used as input for the subdomains were updated in the LCA part. Preliminary sensitivities indicated that this is sufficient at this stage, but it should be noted that the total projected GWP reduction distribution might be slightly too narrow. The model was analysed to estimate potential market penetration and to compute total global reduction of GWP (as carbon dioxide equivalents).

The following section presents a short overview of the methods and building blocks used in the TEA, including 1) LCA, 2) cost analysis, 3) public acceptance, and 4) market penetration model.

### Life cycle assessment (LCA)

An LCA is one of the most commonly used and recognised methods for product system environmental assessments. It offers the possibility to quantitatively compile and evaluate the potential environmental impact of products, technologies, and services from a life cycle perspective [20, 21]. LCA has frequently been applied to assess the environmental sustainability of cement and concrete technologies [22, 23]. It has also been identified as a potential evaluation method for bio-cementation processes [24].

An LCA needs a functional unit to provide a reference for analysis and comparisons based on the product’s capacity to fulfil a function. Here, the functional unit is *1 ton BioZEment*. The scope of the LCA is cradle-to-gate, including the production processes of required materials and energy and emissions from the BioZEment production. Inventory data and descriptions of data sources and assumptions can be found in Røyne [25]. Uncertainty regarding amounts and materials required, emissions from the BioZEment production, and variance between available datasets is accounted for by calculating upper and lower bounds for the impact levels based on ‘best case’ and ‘worst case’ scenarios. The production of peptone and bacteria are omitted due to lack of reliable datasets. The amounts required are small, and the exclusion is therefore assumed not to impact the results significantly. Because of the high uncertainty of water demand at this stage of the BioZEment development, both the GWP of water processing and waste water treatment are omitted. The urea demand has been adjusted upwards and the direct CO_2_ emissions caused by the BioZEment production adjusted downwards in retrospect of Røyne [25]. Oil and cleaner fluid for maintenance purposes were added to the BioZEment production process in the present study (see Table 2) but were excluded from the LCA due to the insignificant amounts. The environmental impact categories of GWP, ozone depletion potential, acidification potential, eutrophication potential, and land use were assessed in Røyne [25]. Based on relative total impact, only GWP is integrated in the TEA and compared with a conventional Portland cement-based concrete product of a similar strength class (C8/10) [26].

**Table 2:** Main influencing assumptions for the analysis. The rest value is 35% stone, 40% sand, and 10% water, resulting in a W/C relationship of 0.65 (base). p10, p50, and p90 represent the 10, 50, and 90 percentiles in the underlying distribution for each assumption. The remaining material, to account for 1 ton of fabricated material, is taken as sand.

Item:	Unit:	P10	P50	P90
**Materials for BioZEment-based concrete**				
Limestone	kg/ton	100	140	200
Urea	kg/ton	2.8	3.4	4.2
Glucose (in bacterial culture medium)	kg/ton	1.0	1.4	2.0
Yeast extract (in bacterial culture medium), dry matter	kg/ton	0.030	0.045	0.070
Peptone (in bacterial culture medium)	kg/ton	0.10	0.13	0.20
Salts (in bacterial culture medium)	kg/ton	0.10	0.13	0.20
Bacterial mass, dry matter	g/ton	2	3	5
Electricity	kWh/ton	3	5	10
Oil	l/ton	0.03	0.05	0.10
Cleaner fluid	l/ton	0.005	0.010	0.020
**Production estimates**				
Number of injections per batch	-	5	10	20
Time between injections	h	1	2	4
Downtime between sets	h	0.1	0.2	0.5
Availability	%	90%	95%	98%
**Market assumptions**		** **	** **	** **
Size of market for concrete	Gigaton	20.2	22.4	24.6
Total share of brick concrete	%	0.15	0.20	0.24
Total share of onsite concrete	%	0.18	0.25	0.29
Total share of prefab concrete	%	0.18	0.25	0.29
Fully developed competitors	-	2	3	5
**Global warming potential**		** **	** **	** **
GWP BioZEment (LCA result; see 3.1)	kg/ton	12.5	17.0	22.6
GWP traditional concrete	kg/ton	70.0	75.6	115.6
**General uncertainty**				
Project write off time	years	7	10	17
Company insurances	%	0.8%	1.0%	1.5%
Company R&D	%	4%	5%	7%
Known unknowns	%	8%	10%	15%
**Public acceptance**				
Change in price point, willingness to pay for ‘green’ solution	%	75%	83%	90%
Change in price point, reluctance to pay for perceived danger, etc.	%	105%	110%	115%

### Cost analysis

Cost input and assumptions are assumed to be uncertain in nature, and thus their predictions in an analysis are not deterministic but probabilistic. In probabilistic reasoning one important concept is hedging one’s uncertainty in a portfolio type of structure. According to Lichtenberg’s successive principle [27], the results of the analysis should consist of a sum of several underlying predictions, where the sensitivity to each underlying prediction should not be dominated by a few large contributions but spread out on several smaller components. The implication is that the uncertainty in the underlying predictions is hedged to reduce the combined uncertainty of all the predictions.

The cost analysis received input from a database with unit cost, and all relevant assumptions are listed in S1 Appendix. The input databases (company databases of Pure Logic AS) are subject to form fitting with distribution functions (Eq 1). Standard triple-goal estimates are used for assumptions with limited underlying data, and these estimates are calibrated with estimation theory (90% confidence interval questions and binary calibration tests) to achieve estimates that are as close as possible to 10%, 50%, and 90% confidence levels [28].

**Fig 1:**
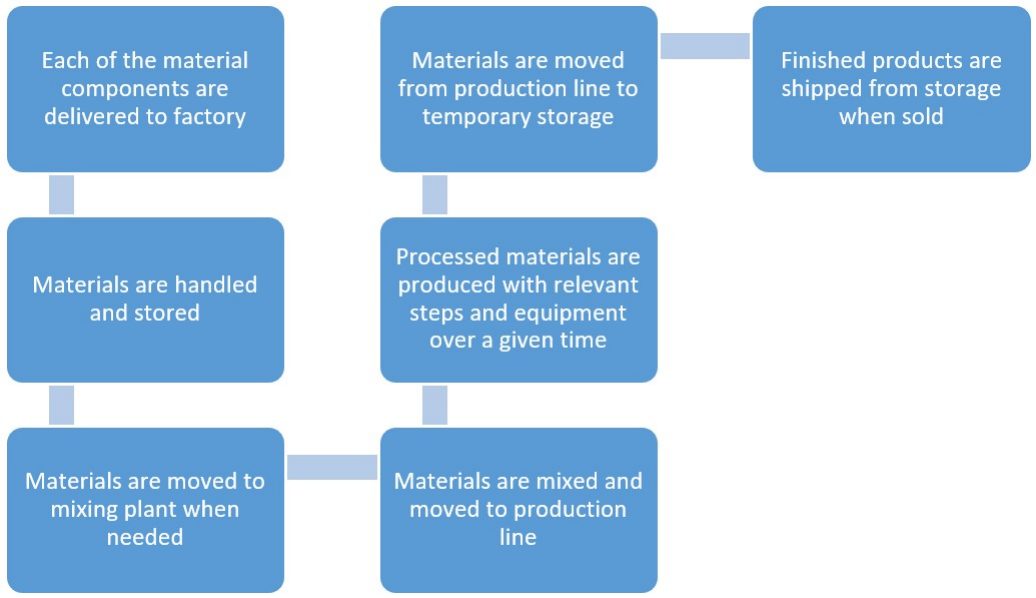
Illustration of a generic production line. Some adaptions to each scenario with respect to different machinery and packing were applied.

Independent unit cost(s) are then combined through logics and arithmetic to form processes. A complete analysis consists of several dependent processes. The dependencies are formed based on causal relations to ensure consistency and to capture non-linear effects from delays or down-time in process lines, as illustrated in Fig 1. Note that only the causal links in production are shown. As the properties of the factory are unknown, the work force, sales force, administration, size, and dimensions of the factory are linked to the production assumptions with estimated distributions (see S1 Appendix for further details).

### Public acceptance

Public acceptance issues have become a primary concern in innovation efforts and have become imperative to ensure product adaption. In general, there are societal concerns over innovations based on emerging technologies [29], including biotechnology [30]. The latter is especially relevant to the scope of this study, where bacteria play a central role. A number of studies on both bacteria in general and on the use of bacteria in products show that some general public concerns exist [31]. Such concerns have over the last decades been particularly visible in the debate on the use of GMOs in food–the ‘GM Controversy’ [32, 33]. Also, a predominant perception of bacteria as a threat to human life, such as related to spreading antibiotic resistance among pathogenic bacteria [34, 35], may partly overshadow the omnipresent, historical, productive uses of biotechnology and the existence of beneficial microorganisms in products used in everyday life.

A general challenge has been to convert the qualitative data and thinking associated with the observed public concerns, and the respective findings in the literature, into numbers that can be integrated into the TEA. A limited literature review on willingness to pay for sustainable consumer products was performed, and the qualitative data points found were used as a basis for estimates that reflect the potential hesitations consumers might have towards buying BioZEment-based products due to (potential GM) bacteria being employed. As there is limited data available at this point of the BioZement development, two high-level estimates were used in the analysis: 1) willingness to pay more for a ‘greener’ product and 2) willingness to buy a cheaper product although possibly perceived as risky.

### Techno-economic analysis (TEA) and estimating input

The TEA is focused on capturing the effects that govern revenue streams and cost drivers. Revenue streams and cost drivers are identified with inductive inference, where existing data are used to generalise the specific problem. Inductive inference is inherently uncertain, as discussed by Gilboa [36]. We estimate the quantitative input data (see also section 2.7) based on the Dempster-Shafer theory [37] and fit the data to the three-parameter lognormal distribution function proposed by Munro and Wixley (1970) [38]:

$$\begin{array}{c} f\left(x\right)=\frac{1}{\sqrt{2\pi }\cdot \sigma \left(1+\frac{\lambda \left(x-\mu \right)}{\sigma }\right)}\cdot \exp\left(-\frac{\log{\left(1+\lambda \left(\frac{x-\mu }{\sigma }\right)\right)}^{2}}{2\lambda^{2}}\right)\\ \sigma >0,\;\lambda \neq 0 \end{array}$$ Eq 1

The resulting uncertainty is managed identically to how risk is managed in the modern portfolio theory proposed by Markowitz in 1952 [39]. The analysis is refined by breaking down dominating assumptions into parts until no single input contributes with more than 10%, to ensure that the portfolio theory [39] still holds.

When input parameters are based on empirical data, it must be asked whether the underlying process of the current problem is the same as the process underlying the empirical data. Differences in scale must be evaluated to assess whether the problem evaluated falls outside the dataset used to estimate the outcome of the current process. In this study, the evaluation of causal relations between assumptions and activities is used to analyse the relevant processes and divide them into sub-processes that can be assessed and estimated with special care regarding validity restrictions.

**Fig 2:**
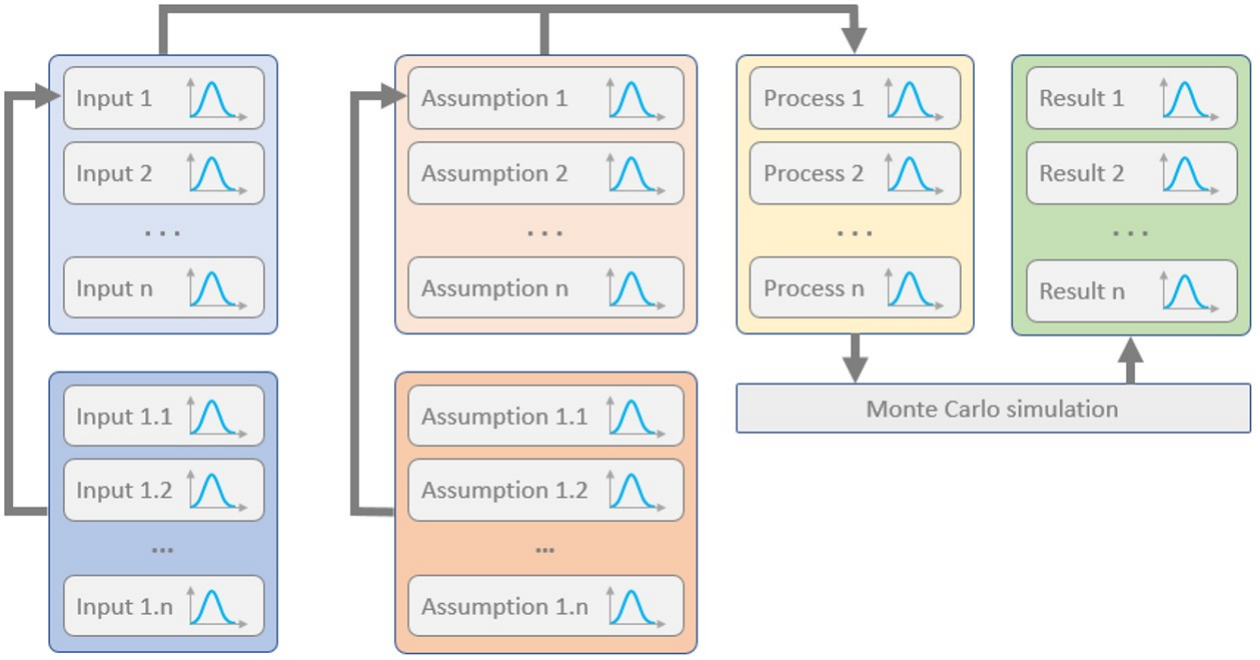
General flow-chart of input costs and assumptions handling in the TEA. A complete list of the inputs and assumptions is shown in S1 Appendix.

To ensure that all relevant assumptions and costs are handled appropriately, the successive principle suggested by Lichtenberg [27] was applied with a top-down approach and calibration with known products in each of the scenarios. The estimates and possible outcome scenarios were formed using the strategies suggested by Tetlock and Schoemaker [40], with special focus on avoiding cognitive biases and the following three-step scenario evaluation: 1) diverging, 2) evaluation, and 3) converging. To maintain control of the influencing uncertainties, a multi-perspective scenario evaluation [40] was then used to uncover potential implications. A simplified flow-chart of the setup is shown in Fig 2. Inputs and assumptions are listed in section 2.7 and S1 Appendix.

A distribution of output values was generated through a standard Monte Carlo approach [41], where the output is a result based on a set of random values drawn from the distributions of each of the input values that are combined in the modelled activities. The distribution of output values is typically found to converge after around 1 000 realisations. The output values calculated in this study are unit price and GWP reduction.

A sensitivity study was applied to the output to identify the most influential assumptions. This can be used to indicate possible areas of focus for further development and to ensure that there are no parameters that dominate solely.

### Market penetration model

**Fig 3:**
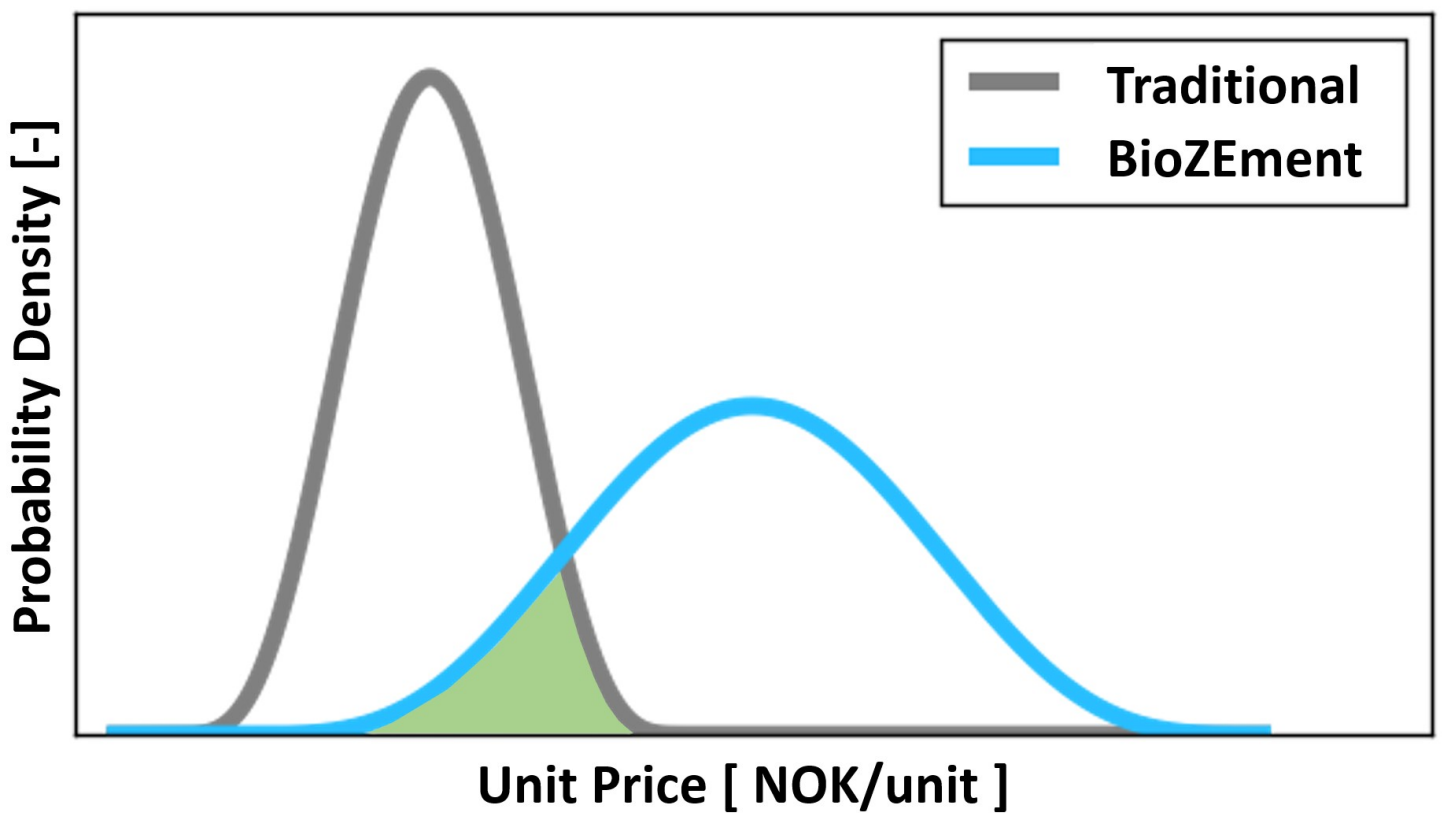
Illustration of the total market segment, shown in green, where BioZEment-based products are assumed to compete (rational substitutes) with traditional products.

For each of the three scenarios of potential BioZEment applications listed in Table 1, a probability distribution of unit prices was generated using a Monte Carlo approach on the TEA. For each Monte Carlo realisation, the resulting price of the BioZEment-based product in question was compared to the price distribution for traditional concrete to identify its percentile location. The price of BioZEment was computed with the relevant distribution for cost, reluctance to buy the novel product, and the increased willingness to pay for lower greenhouse gas emission with the input assumptions, as shown in S1 Appendix. In each realisation, the BioZEment-based product was then assumed to be a potential substitute for all customers buying more expensive concrete in the current scenario. The market volume was assumed to be distributed similar to the price distribution. Combined, this yielded the total market segment where the BioZEment was competing. Further, the market was assumed to be split equally into a distribution of market actors estimated to be within this segment. The distribution for the total market share of BioZEment-based concrete follows directly from these assumptions. An idealised illustration of the total market share where the BioZEment can compete with traditional products is presented in Fig 3.

### Investigated scenarios

#### Brick production

For brick production, we assume a large factory with a semi-automated production line based on Revaro’s setup [42]. The production line is assumed to be dependent on manual labour. The factory is only capable of producing standard, massive concrete stones. We assume it is not possible to omit the injection process described in [4]. The number of injections of bacteria and nutrients, time between injections, and production area are the limiting factors in the production line [4]. Production line assumptions comprise a) a theoretical capacity per set of 10,640 stones/bricks per set/cast (10 blenders à 8 m^3^, 20 founders à 16 m^2^) and b) a production line mix of standard massive concrete stone/brick of the dimensions of H = 6 cm, W = 10,4 cm, and L = 26 cm. We further assume a simple production line, highly dependent on manual labour, located in a region with high wages.

#### Precast production

We assume a large factory with a fully automated production line based on Elematic’s setup [43], reliant on standard production equipment from an external supplier. The factory can produce different types of hollow core slabs. In addition, the slabs can be tailored right on the casting bed (place recesses, holes, and other openings). We assume it is not possible to omit the injection process described in [4]. The number of injections, time between injections and production area are the limiting factors in the production line. Production line assumptions comprise a) a theoretical daily capacity of 1,435 m^2^ per set/cast (beds: 8 pcs, width: 1.2 m, length: 150 m) with a size of the production line that is comparable to a large, modern factory in Europe and b) a production line mix of 25% HD200, 25% HD265, 25% HD300, and 25% HD 400. As simplifications, we assume a) that the BioZEment technology is mature and that batching and mixing are comparable with today’s approach: mixed BioZEment-based concrete is sent from the batching and mixing plant via shuttles to production beds distributors, and injections continue on the casting bed; and b) a static production line mix that is not optimised to increase earnings (based on market demand).

#### Onsite production

Onsite production of BioZEment-based concrete is assumed for building the floor and walls on a generic building, where formwork is the most time-consuming part of the operation. Here, we assume that it is possible to omit the injection process described in [4]. In this scenario, BioZEment-based concrete is used to build a generic 5 000 m^2^ building (floor and walls), including formwork and reinforcement (fibre or ducted steel reinforcement). The amount of BioZEment-based concrete needed is assumed to be around 2 500 m^3^ (assuming square floor with a thickness of 0.4 m, outer wall with a height of 3 m and wall thickness of 0.3 m, and corresponding amount of inner wall as outer wall).

### General limits and assumptions

Each of the scenarios assume that we are able to obtain mechanical properties similar to those the product categories represent, something which has not yet been fully technically proven [4]. It is also presumed that the introduction of BioZEment-based products does not change the total number of products in each scenario (static demand). The investigated scenarios adapt a typical production scheme, with respect to automation. Any change in automation level is assumed to be applicable for the production of both BioZEment-based and traditional products. Thus, the production volume may change, but the difference between the standard and the BioZEment product will remain somewhat the same. This allows for the modelling of only one type of production line per scenario.

Only a regional cost database (a compiled database with index regulated prices to 2017 NOK; the base consists of Norsk Prisbok 2017 [44], the internal databases of Pure Logic AS, and data collected from various suppliers and distributors) is applied in the analysis, while the scoped projection is global. The distributions used in the analysis are listed in S1 Appendix. The ratio between material and labour costs will vary significantly between regions, but as long as this is mostly a substitute to traditional concrete, it can be assumed to scale well with regional differences for traditional concrete. This has not been proven and should be an important item for further work.

The LCA is uncoupled from the dynamic cost analysis, using only a three-point estimate. We assume that this has a limited effect on the results, compared to the other simplifications made, and that the influence is mainly on the width of the uncertainty of GWP reduction rather than the expected value.

The cost module and market analysis further assume a fully developed market, with established actors with identical profit margins and similar market shares. At the current stage, the total revenue is taken as market size as there is a relatively small difference in cost for large volume concrete production (the cost difference for low strength vs high strength concrete is in the range of 10%; the product price difference for complex concrete products may be significantly higher, but this is due to secondary effects such as higher requirements for reinforcement, challenges in handling, etc.), and only a limited differentiation of products (for prefabricated elements only) is performed. The material is assumed to have been developed to a level where it is perceived as safe by customers and/or consumers.

Regional, national, and/or global regulations have not been accounted or adjusted for. This includes the exclusion of any CO_2_ taxing schemes as they were found to currently have limited to no significant impact on the predicted cost of the product.

Any handling or costs related to waste are omitted.

A complete list of the assumptions for each scenario is shown in S1 Appendix, but the most important are shown in Table 2.

## Results and discussion

### LCA

The LCA result is presented in Table 3 both as absolute values and as a percentage distribution between the production of the required materials and electricity for the production of BioZEment-based concrete and the BioZEment production process itself. The variation between the lowest and highest estimate, in total results and for the individual processes, reflects both the technical uncertainties and the variations in life cycle inventory databases. The GWP values are collected from databases. The value for urea was calculated based on several sources. The Ecoinvent dataset [45] gives a very high climate impact per kg urea (3.3 kg CO_2_-eq). When compared with the compilation of greenhouse gas emission factors by [46], it appears that the high impact of the Ecoinvent dataset is due to the reference to nitrogen (‘as N’). Alternatively, [46] present one value ‘per kg N’ and one ‘per kg product (urea)’. Since the relation between the ‘per kg N’ and ‘per kg product’ of [46] is 2.17 between all the four references to urea, we chose to divide the impact in the Ecoinvent dataset by 2.17.

**Table 3:** GWP of the production of 1 ton of BioZEment-based concrete.

Production process	Assumptions		GWP
	Lowest estimate	Highest estimate	
Limestone	5 μm grain size [47]	50 μm grain size [48]	20–29%
Sand [49]	One scenario		14–25%
Electricity	Norwegian electricity mix [50]	European electricity mix [51]	2–10%
Urea	2.8 kg demand	4.2 kg demand	28–34%
Glucose	Sugarcane and 1 kg demand [52]	Sugar beet and 2 kg demand [53]	2–4%
Yeast [54]	0.03 kg demand	0.07 kg demand	<1%
Salt [55]	0.1 kg demand	0.2 kg demand	<1%
BioZEment	One scenario. Fossil CO_2_ from the hydrolysis of urea (fermentation of glucose produces biogenic CO_2_). One mole of hydrolysed urea results in two moles of CO_2_		14–16%
SUM			12.5–22.6 kg CO_2_-eq

According to the GaBi dataset [26], conventional Portland cement-based concrete of a similar strength class (C8/10) has a GWP of 75.6 kg CO_2_-eq/ton. BioZEment has 70–83% lower GWP than the conventional concrete and is thus in line with the aim of developing a material with superior climate performance. Ideally, in addition to the strength class, the mechanical properties of the BioZEment should be reflected in the comparison. However, these have not yet been tested as the BioZEment development has not yet reached a definite solid product phase. Furthermore, the dataset for conventional concrete represents Chinese production, which might not be the most appropriate to base the comparison on. However, all available strength class defined datasets are based on Chinese production. Datasets were chosen with technical relevance as the primary priority and geographical relevance as the secondary priority. It should also be mentioned that by replacing a part of the cement content in concrete with other materials, such as fly ash, the climate impact of conventional concrete can be reduced. This is, however, possible to a lesser degree for low strength concrete, where the cement amount is already low.

As mentioned in section 2.1., environmental impact categories other than GWP were omitted in this study based on their relative total impact. However, Røyne [25] shows that BioZEment has a higher environmental impact than conventional concrete in the categories of ozone depletion, eutrophication potential, land use, and higher acidification potential. Still, as discussed in Røyne [25], the severity depends on absolute results. Ozone depletion levels were concluded to be insignificant. The significance of acidification and eutrophication levels are more uncertain as no studies could be found that commented on such impact from conventional concrete production. The main impact source from the BioZEment production is ammonia emissions from the BioZEment, resulting from the hydrolysis of urea [4]. Ammonia capture and recycling have been demonstrated in other bio-cementation efforts [6] and will also be considered for BioZEment.

Land use for BioZEment production is up to 6–10 times higher than that of conventional concrete, mainly due to glucose production, but is still less than 1% of that for timber [56]. As stated in section 2.1., water use is not included in the LCA as the necessary requirements have not yet been established. This is a factor that needs further examination as the bio-cementation process in its current state of development requires significant amounts of water, in particular in the multiple injection process. However, given an injection-based process, the water is assumed to be easily recycled.

### Public acceptance

For the time being, natural occurring isolates (bacteria) are used in the BioZEment process. However, an optimisation by genetic engineering is under continuous consideration in the project consortium [4]. This option is left open in the event that the natural isolates do not sufficiently handle process conditions and/or if their production rates of acid or ammonia via hydrolysis by urease are less than desired. Genetically engineered/genetically modified organisms have raised public concern in the past, particularly in Europe [57]. Such concerns, including perception of risks and benefits, eventually influence consumers’ behaviour and decision-making and their acceptance of the technology in question (here: biotechnology/genetic engineering) as well as their willingness to buy novel products based on such technologies [58]. This is the rationale for the attention given to the public acceptance of GMOs in this contribution.

The literature on GMOs is dominated by the potential use of such organisms for food production, and rather few contributions address other potential uses. It is interesting to note that there has been little controversy over the use of GMOs in the production of medicine [59], but somewhat more in, for example clothes [60] [61]. GMOs are by some viewed as unnatural [62], and naturalness is often an important aspect of consumers’ interpretation of sustainable products [63]. An interesting survey, and the only study we have identified that compared GMOs used for different products (food, clothes, and decorative flowers) is published in a report by Bugge and Rosenberg [64]. The respondents were more positive/less negative towards the use of GMOs in flowers and clothes, especially when compared to its use in food. However, of particular interest here was that the survey respondents stated that in general they were more concerned about the potential negative effects on the environment than about their own health [64].

Studies of willingness to pay usually include surveys in which the authors have asked respondents specifically, but with slightly different wording, how much they are willing to pay for ‘green’, sustainable products [65, 66, 67]. Two of the studies reviewed, one on the willingness to pay for eco-friendly products [67] and the other on the willingness to pay for certified wood products [68], estimate the willingness to pay more for sustainable products to be 10%. However, this number may be even higher, as studies have found a significant variation in consumers’ levels of awareness and willingness to select higher-priced environmentally friendly products [66]. One reason why consumers would be willing to pay a premium is product quality, which could be interpreted in two ways: objective quality, measurable relative to a standard product, and perceived quality, in the judgement of the individual consumers [65]. In the study involving certified wood [68], it was estimated that the maximum price premium, given specific-stated preferences (e.g. trust in labelling schemes, having bought other labelled products, higher annual income), is not larger than 25%.

Based on these two studies, we estimate the willingness to pay a price premium to be in the range of 10 to 25% (p10, p90). We have not found similar studies on willingness to pay, or reduced willingness to pay, for GMOs. However, at least among European and Norwegian consumers, there still appears to be a reluctance and scepticism towards such products. Building public trust in the institutions involved in research and innovation development is crucial for the evaluation of biotechnology-based products in specific, but even for biotechnology in general [58]. It is hard to assess the effects on the willingness to pay, but it appears reasonable to say it will push the price premium to the lower end of the range. In the analysis, this is done with an individual estimate for a reduced price point of 5–15% (p10, p90). Transparency in the further development and eventual marketing of BioZEment-based products could assist in pushing the premium price higher up in this range.

### Cost analysis

**Fig 4:**
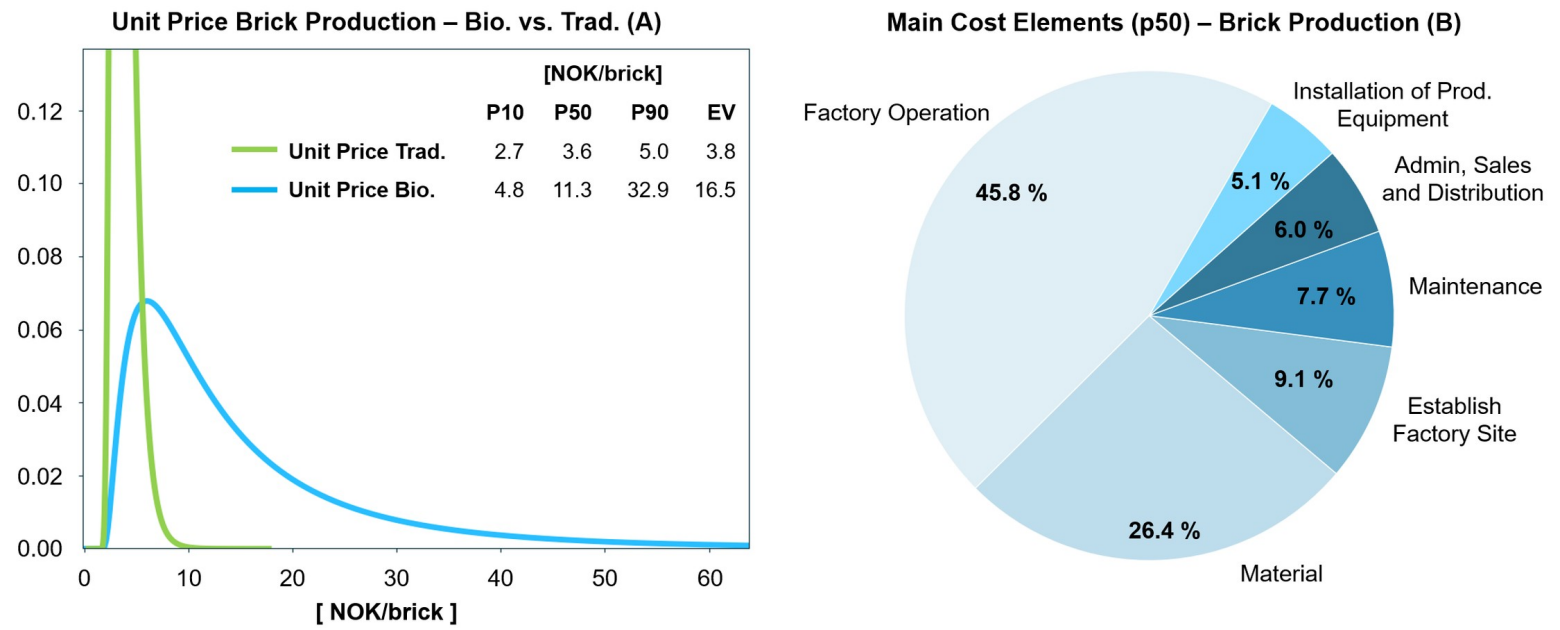
(A) Comparison of the estimated unit price distribution of traditional versus BioZEment-based brick production (blue); (B) main cost elements at p50 percentile.

Cost analyses were performed for each of the three scenarios. The results are summarised in Fig 4 to Fig 9. The distributions of expected unit price for a Portland cement-based product and a BioZEment-based product are shown in green and blue, respectively. The main cost elements (for the most likely scenario, p50, in the distribution) are also shown. In addition, a sensitivity analysis is shown comparing the importance of input uncertainties in terms of their relative contributions to uncertainty on the output (unit price).

For each of the scenarios, the material price alone is about 10% higher for BioZEment than for conventional concrete. To reduce cost, it is therefore important to focus on developing more efficient production methods, in particular through reducing the number of injections. This is particularly critical for the brick and precast scenarios.

**Fig 5:**
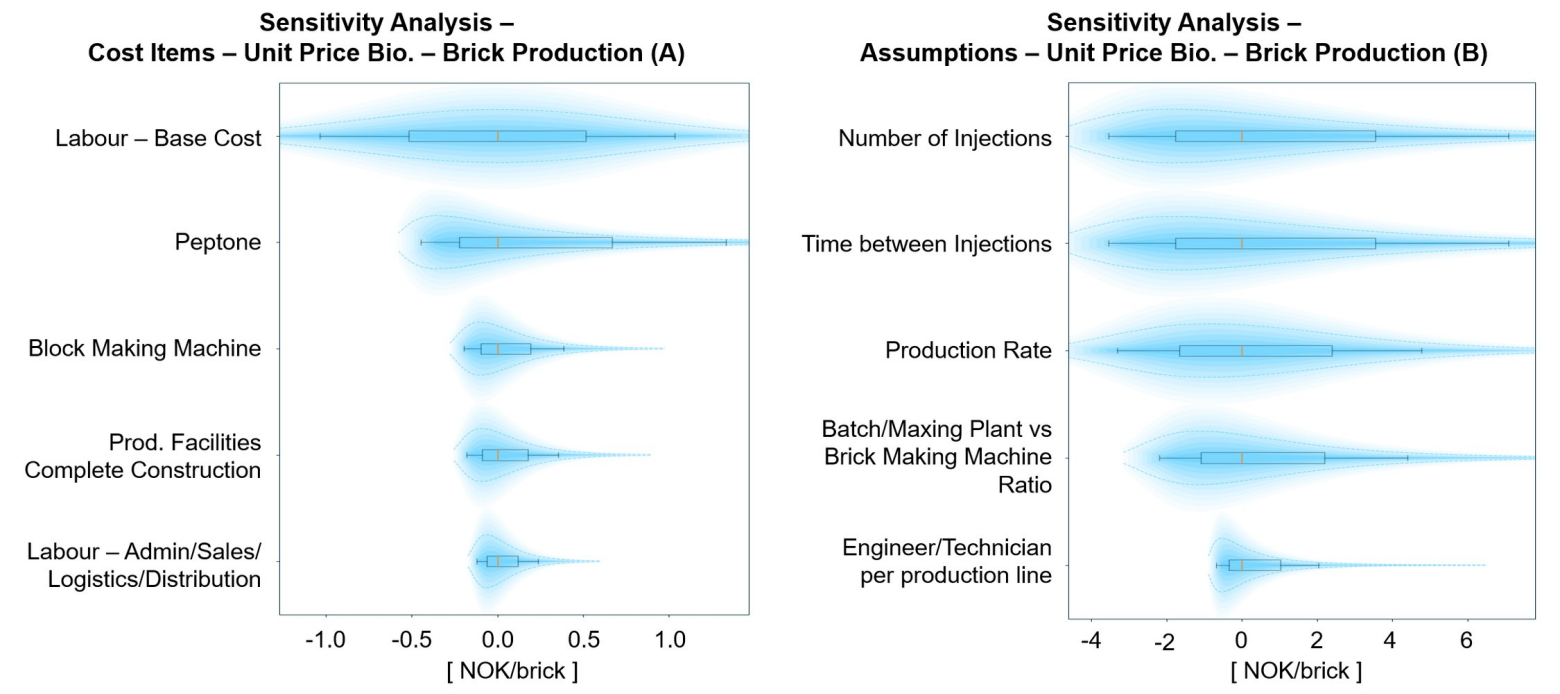
Probabilistic sensitivity analysis on cost items (A) and assumptions (B) for the unit price of BioZEment-based brick production. The plots show the top five elements with the largest impact on the output, keeping all other input variables at p50 percentile. Each element is shown with p10, p30, p50, p70, and p90 percentiles (illustrated as box plots), including their respective distribution.

**Fig 6:**
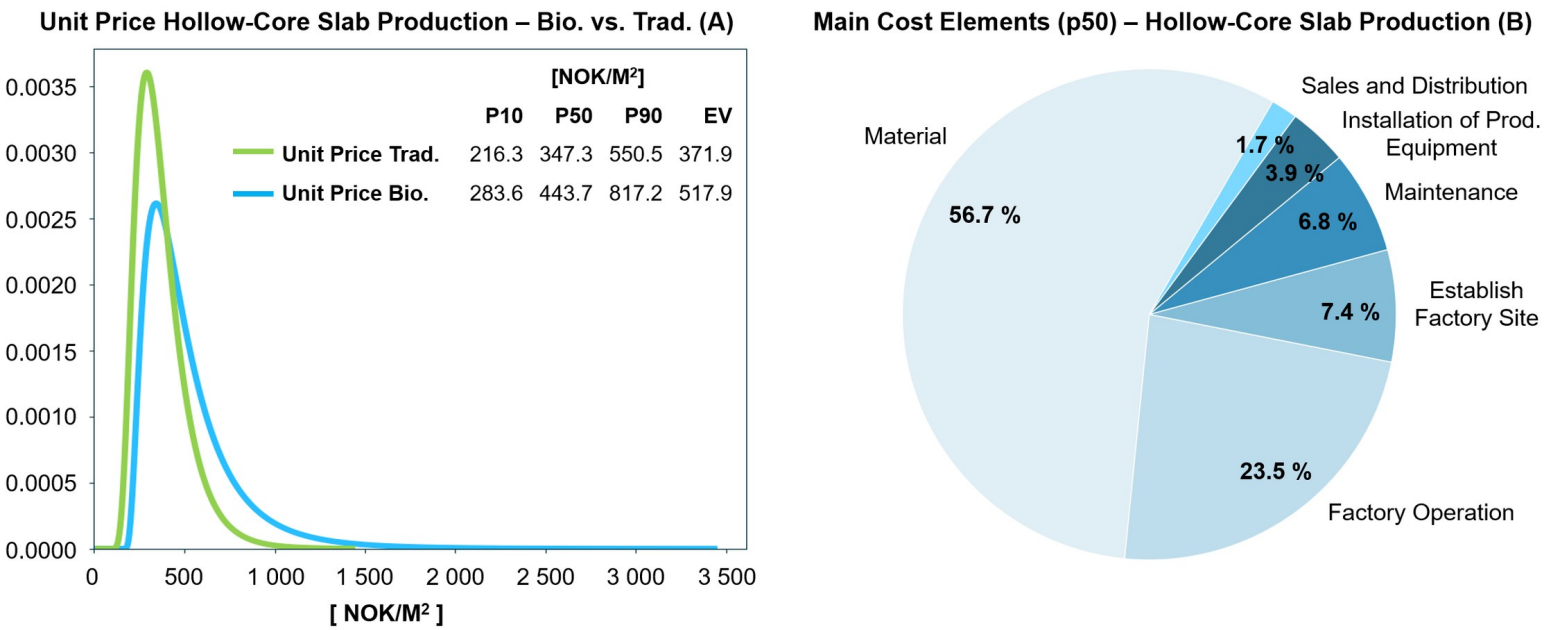
(A) Comparison of the estimated unit price distribution of traditional versus BioZEment-based precast production (blue); (B) main cost elements at p50 percentile.

**Fig 7:**
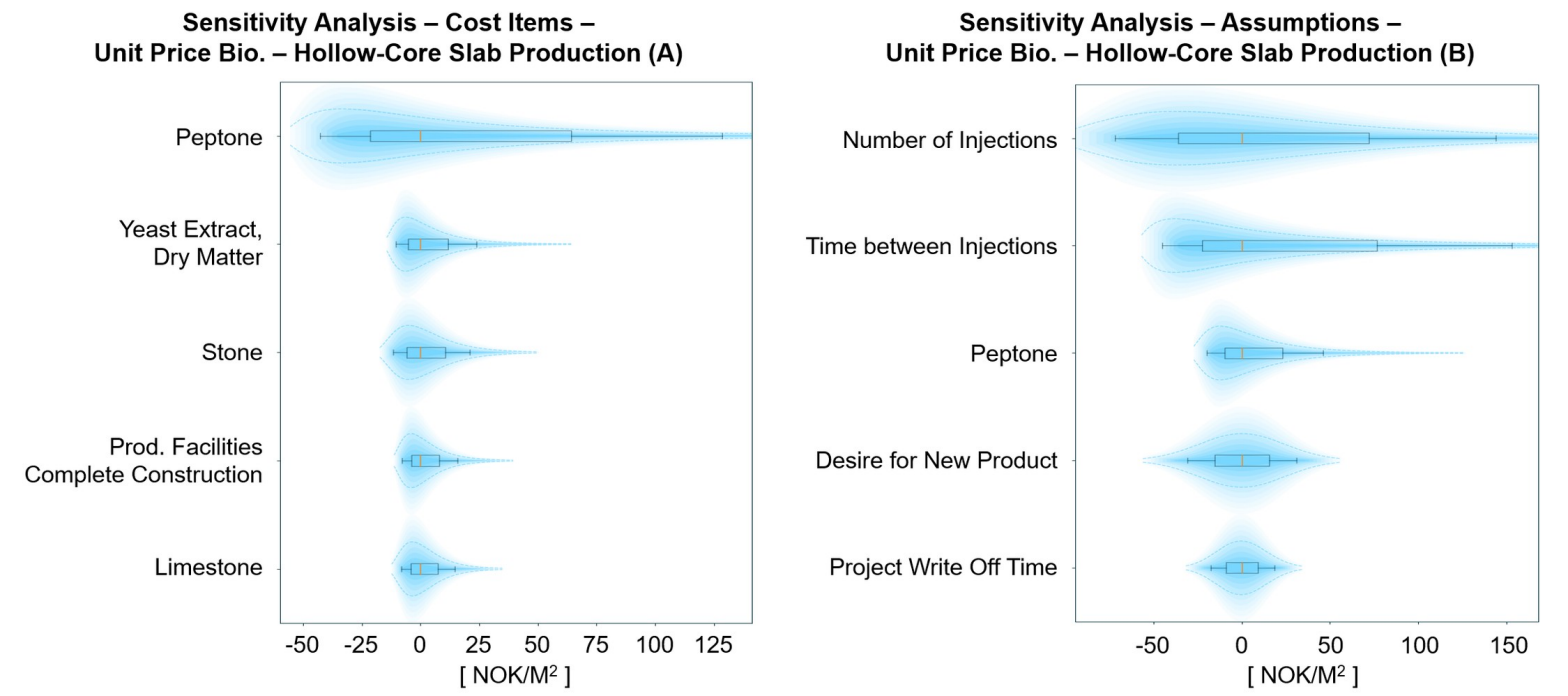
Probabilistic sensitivity analysis of cost items (A) and assumptions (B) for the unit price of BioZEment-based precast production. The plot shows the top five elements with the largest impact on the output, keeping all other input variables at p50 percentile. Each element is shown with p10, p30, p50, p70, and p90 percentiles, including their respective distribution.

**Fig 8:**
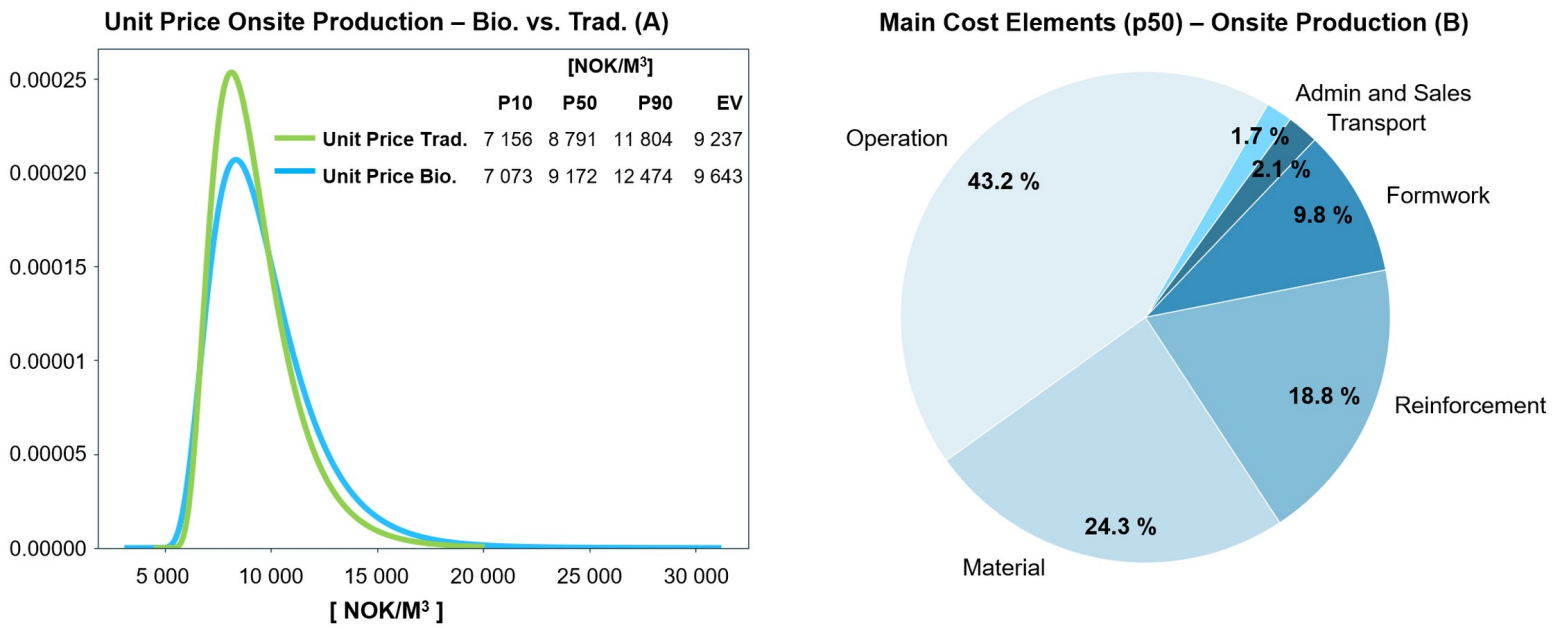
Fig (A) compares the estimated unit price distribution of traditional versus BioZEment-based onsite production, while Fig (B) shows the main cost elements at p50 percentile.

**Fig 9:**
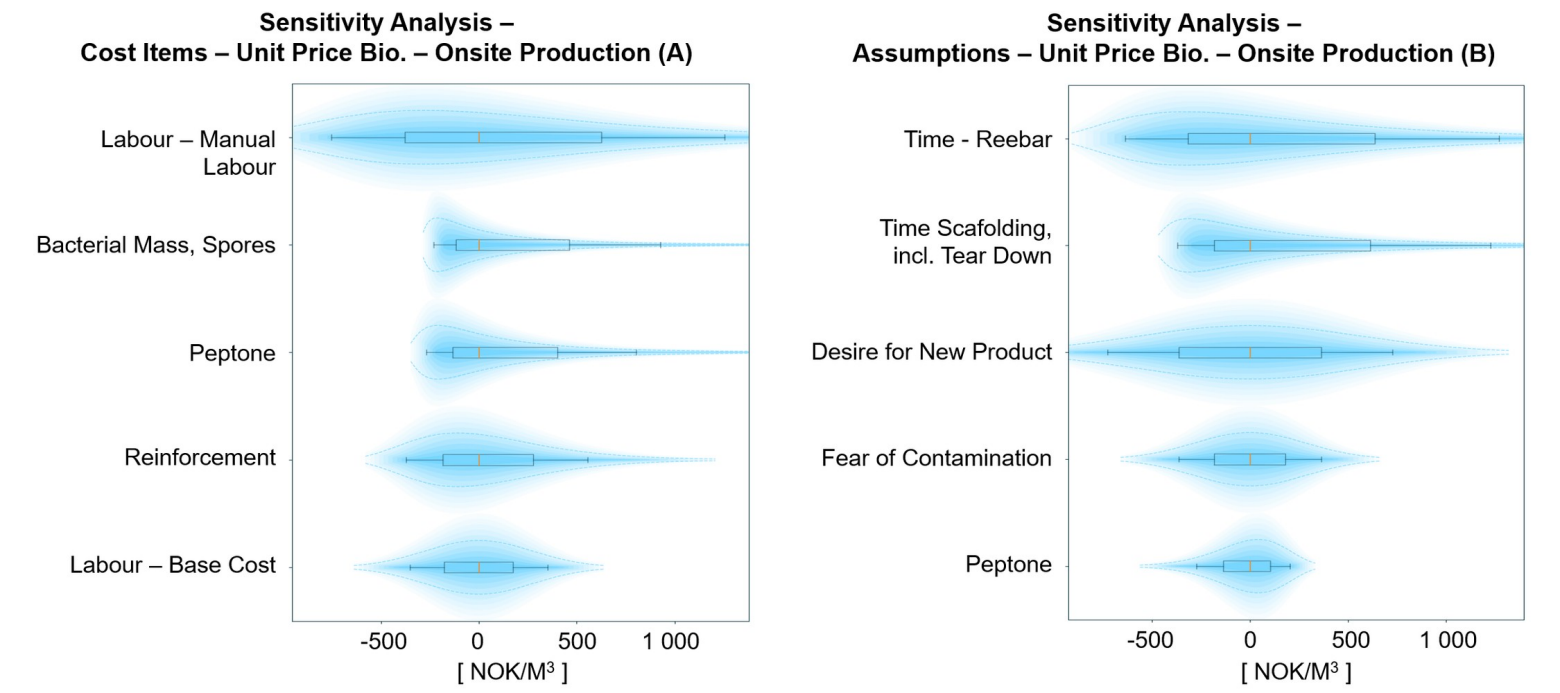
Probabilistic sensitivity analysis on cost items (A) and assumptions (B) for the unit price of BioZEment-based onsite production. Shows top five elements with the largest impact on the output, keeping all other input variables at p50 percentile. Each element is shown with p10, p30, p50, p70, and p90 percentiles, including their respective distribution.

#### Brick production

BioZEment-based brick production will be more expensive than traditional brick production, as shown in Fig 4A. This is mainly due to production speed (injection phase), which is considerably lower compared to the traditional production of bricks. As shown in Fig 5, the dominating uncertainties are at the current stage related to the injections, and technical improvements in this area could lower the unit price significantly. Without improvements, it will be hard for the BioZEment brick to compete with the mass production of conventional traditional bricks. However, BioZEment-based bricks might be viable as a niche to enter the market and grow a brand. Production can be more automated to take down the costs further. General progress on automation is not thought to be more favourable for BioZEment-based bricks than traditional bricks, as the total ratio of machinery and infrastructure is likely to remain similar.

#### Precast production

The production of BioZEment-based hollow-core slabs can compete directly with traditional production (Fig 6A) and is highly dependent on the curing time of BioZEment compared to traditional. Compared to brick production, curing time is not as critical for the precast production.

The production is assumed to be fully automated, where material cost comprises the largest portion when breaking down the unit price (Fig 6B). If the production setup results in lower curing times for BioZEment compared to traditional, then BioZEment will have good opportunities to take a large market share. The uncertainty in the unit price of BioZEment-based precast production lies in injection-related assumptions as well as the cost of peptone (Fig 7).

#### Onsite production

When assuming no injection in producing BioZEment-based concrete onsite, comparable process results at a comparable price level of traditional concrete are obtained (Fig 8A). Some of the differences in price can be explained by the difference in material. The main cost components for producing onsite concrete are related to direct labour, as shown in Fig 8B.

Several of the uncertainties in the assumptions shown in Fig 9 also apply for traditional production. Besides assumptions that are equivalent for both, the uncertainty in the unit price of onsite-based BioZEment products lies mainly in the balance between the market’s desire and potential for negative reaction by the consumers when introducing products based on novel technologies.

### Market penetration and reduction of environmental footprint

The market penetration potential is performed on the assumption that the BioZEment will compete with well-established producers of conventional concrete. The following results show the predicted GWP reduction for each of the investigated scenarios.

The market share and equivalent reduction in environmental footprint is computed separately for each of the scenarios. As each of them have independent project constraints, it is not relevant to evaluate them with respect to the uncertainties and probability of successfully being able to fully comply with the assumptions. The results should be taken as the expected potential, given that the main constraints for each scenario are solved.

### Brick production

The total market share for BioZEment-based brick products is found to be between 0.0 and 4.5% (p10, p90), with the most probable value (p50) of 0.0%. For the p50 percentile, this results in negligible total reduction in GWP. This means that the estimated price difference between BioZEment and traditional bricks is so large that consumers will most likely not choose to buy BioZEment bricks instead of traditional bricks, at least not in a significant amount. Due to the large price difference, and the estimated p50 market share at zero, there is no purpose in showing the sensitivities for GWP/ CO_2_ reduction.

### Precast production

**Fig 10:**
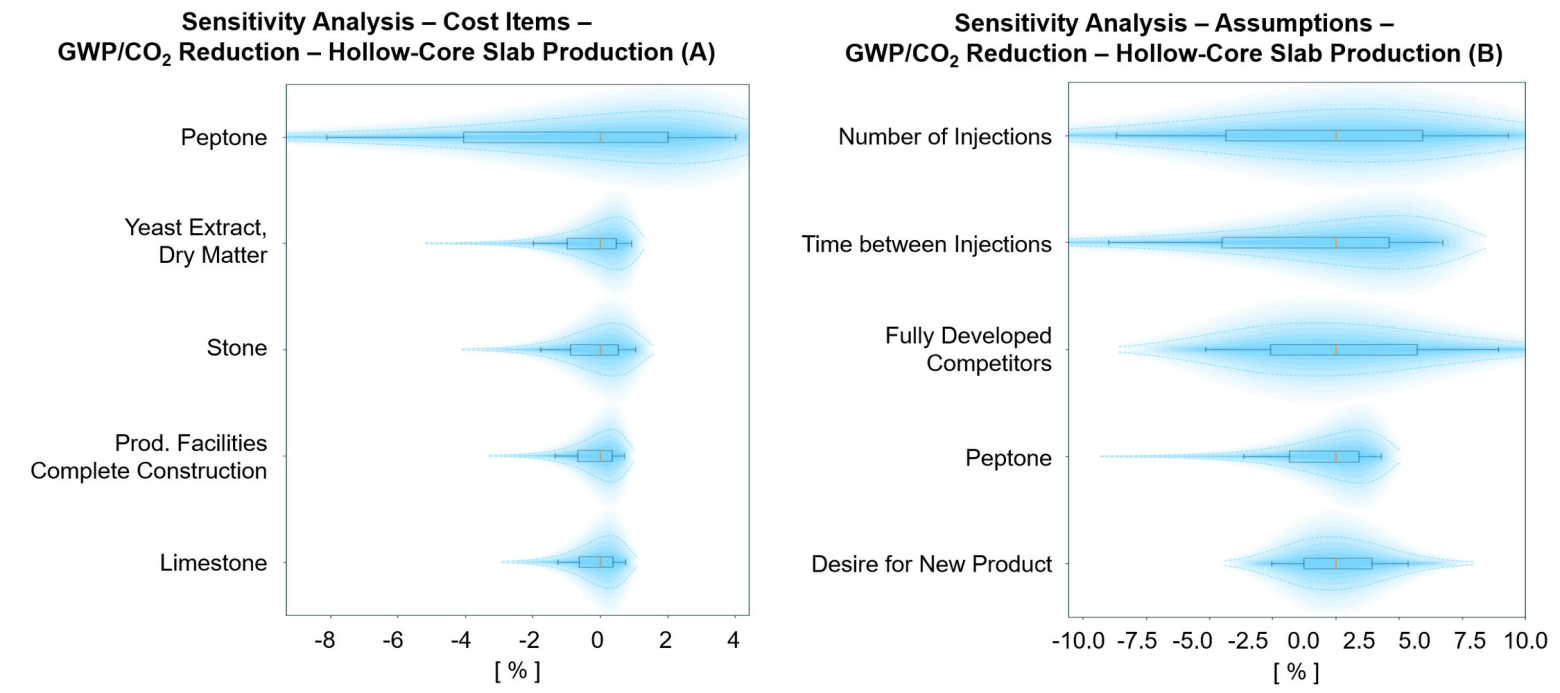
Probabilistic sensitivity analysis on cost items (A) and assumptions (B) for the GWP reduction of BioZEment-based precast production. The top five elements with the largest impact on the output are shown, keeping all other input variables at p50 percentile. Each element is shown with p10, p30, p50, p70 and p90 percentiles, including their respective distribution.

The total market size for BioZEment-based precast products is found to lie between 0.3 and 25.2% with the most probable value of 7.0%. The resulting GWP reduction is calculated to be between 1.1 and 100.7 Megaton CO_2_ equivalents, with the most probable value (p50) of 22.2 Megaton CO_2_ equivalents. The results are most sensitive to the cost of peptone, time between and number of injections, and uncertainties related to reluctance to apply the new product (Fig 10).

### Onsite production

**Fig 11:**
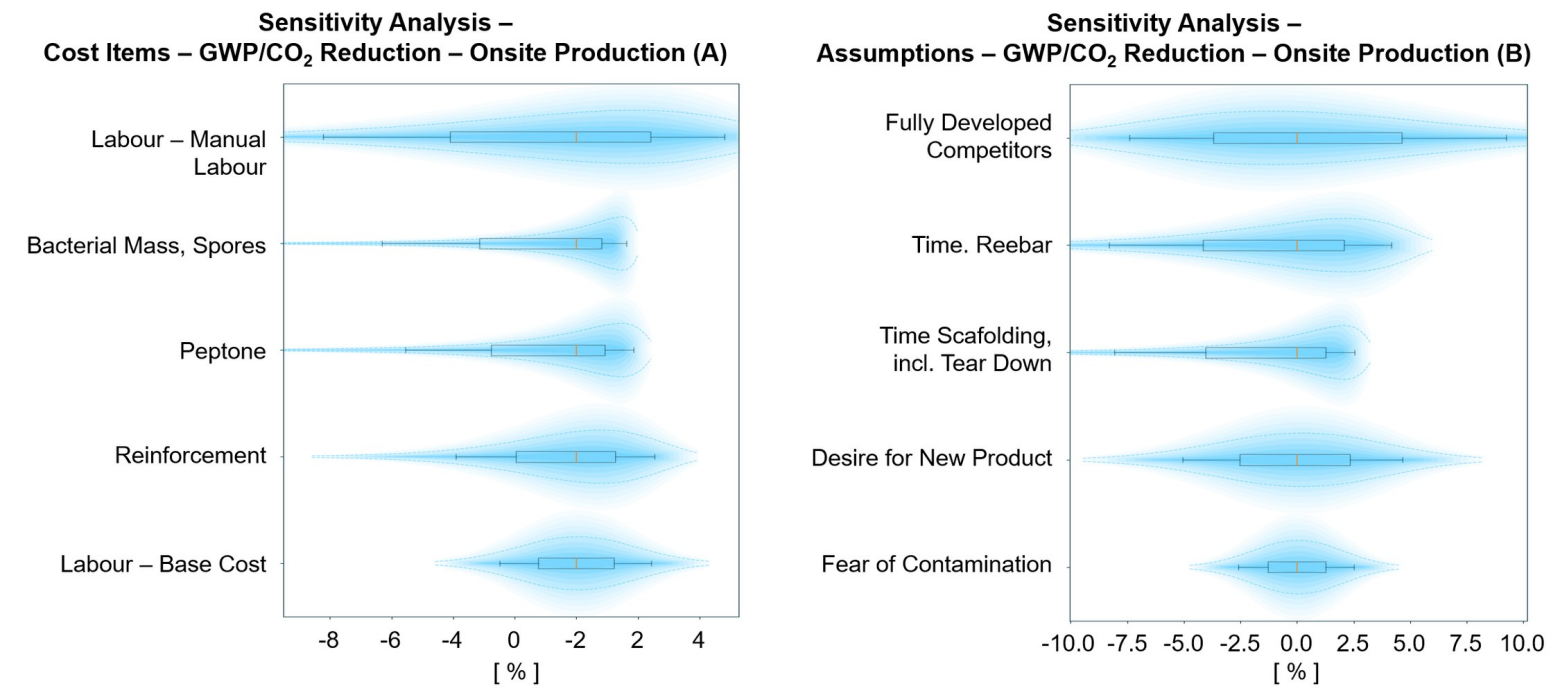
Probabilistic sensitivity analysis on cost items (A) and assumptions (B) for the GWP reduction of BioZEment-based onsite production. The top five elements with the largest impact on the output are shown, keeping all other input variables at p50 percentile. Each element is shown with p10, p30, p50, p70, and p90 percentiles, including their respective distribution.

The total market size of BioZEment-based onsite production is found to be between 1.8 and 32.9% with the most probable value (p50) of 13.5%. The resulting GWP reduction is 5.7 to 130.8 Megaton CO_2_ equivalents (p10, p90), with the most probable value (p50) of 43.1 Megaton CO_2_ equivalents. The results are sensitive to uncertainties related to reluctance to adapt the new product, the labour required for reinforcement and building scaffolding, and worker efficiency. The cost of peptone and uncertainties related to the quantity of bacteria required for this scenario without multiple injections is also significant (Fig 11).

A cheaper product will typically be adopted in an existing market, but it is important to incorporate the consumer risk perception to establish whether the product is perceived to have higher value relative to cost in the end. The results show that although the BioZEment-based products in general are more expensive than conventional concrete, they still have the potential to substitute a significant portion (several %) of traditional products and thereby make a significant reduction in global GWP.

### Implications of public acceptance

**Fig 12:**
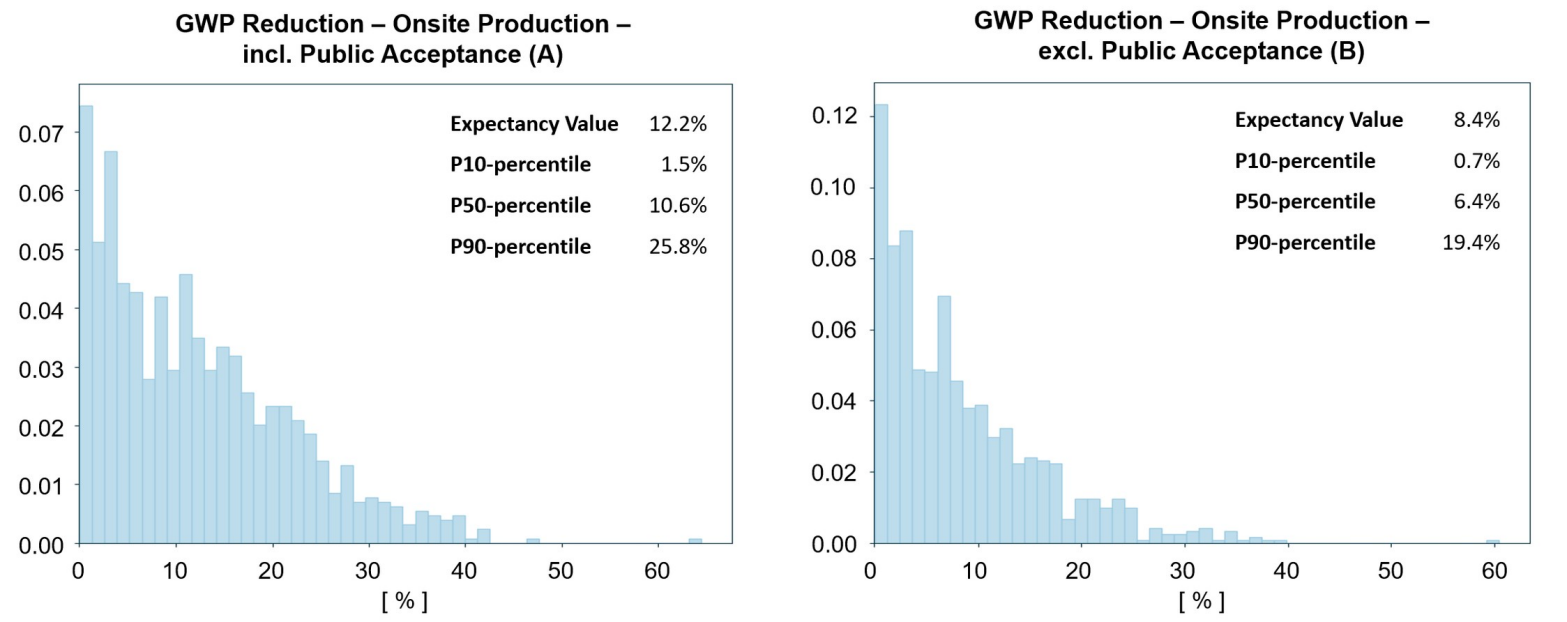
Histograms that chow the percent GWP reduction, with (A) and without (B) taking PA into account for onsite production.

The scenario results show that the analysis is sensitive to the inputs for public acceptance. Table 4 summarises the total estimated GWP reduction with and without accounting for the public acceptance aspect and is illustrated visually for the onsite scenario in Fig 12. With the current assumptions, the expected GWP reduction is increased significantly, especially for onsite production. The highest potential for GWP reduction is in the onsite scenarios, but it is imperative to note that the change in results from these single parameters also implies that more work should be invested in this topic.

**Table 4:** Influence of public acceptance on the total GWP reduction for each scenario. The numbers are given in p10, p50, and p90 percentile for the distribution estimated, including the expectancy value.

	GWP Reduction excl. Public Acceptance				GWP Reduction incl. Public Acceptance			
	p10	p50	p90	Expectancy Value	p10	p50	p90	Expectancy Value
**Brick**	0.0%	0.0%	1.7%	**0.9%**	0.0%	0.0%	3.5%	**1.4%**
**Precast**	0.1%	4.0%	16.0%	**6.2%**	0.3%	5.5%	19.7%	**8.0%**
**Onsite**	0.7%	6.4%	19.4%	**8.4%**	1.5%	10.6%	25.8%	**12.2%**

## Conclusions and outlook

### Potential to compete as a competitive, viable product

The material cost of BioZEment-based concrete is about 10% higher than ready-mixed concrete. The ability to compete on price for a manufactured end-product is highly dependent on the production process and the limitations it may impose. The marginal cost difference on material may be outweighed by an increased willingness to pay for ‘greener’ products. However, material cost is only one of many contributors to the final cost of a complex structure or delivery. For a typical building project, where direct concrete cost accounts for 10–20% of the total cost, use of a BioZEment-based concrete product will increase the total costs by 1–2%. Thus, the influence on the end-product price can be assumed marginal. It should be emphasised that this is under the given limits and simplification of the analysis and that this is not likely to be representative for early prototyping and small-scale production where significant trial and error to establish robust handling and manufacturing processes should be expected.

#### Potential to reduce GWP

The LCA indicates that BioZEment-based products have the potential to reduce climate impact considerably (70–83%) compared to similar strength class conventional concrete.

As shown in Table 5, as cost is somewhat higher than for traditional concrete products, BioZEment products will have to rely on public acceptance-related factors to ensure large-scale use. When accounting for this, the total potential for GWP reduction for each of the three scenarios are as follows:

**Table 5:** Total estimated potential for GWP reduction for brick, precast and onsite production.

	Potential GWP Reduction [Megaton CO_2_ equivalents]			Potential GWP Reduction [% CO_2_ equivalents reduction]		
	p10	p50	p90	p10	p50	p90
**Brick Production**	0.0	0.0	12.0	0.0	0.0	3.5
**Precast Production**	1.1	22.2	100.6	0.3	5.5	19.7
**Onsite Production**	5.6	43.1	130.8	1.5	10.6	25.8

#### Increase potential for GWP reduction

We can identify two important strategies for increasing the potential for GWP reduction:

1. Achieve faster hardening/injection time to obtain fast casting cycles
2. Further understanding of public acceptance

If one could perform the injections quickly, and one were able to reduce the total number of injections needed, it would open the possibility to harden faster than traditional concrete can, resulting in more casts per day. This is of special importance to the precast scenario, which is very sensitive to the number of injections per time unit on the available infrastructure.

The ability to communicate the potential GWP reduction and prove that the product is safe to use is imperative when the cost difference is limited. Increased willingness to pay for the product, when compared to a substitute, is a key driver in the potential market share.

#### Drivers for uncertainty

With respect to assumptions, there are two major drivers of uncertainty. The first is how public acceptance and willingness to pay for BioZEment products affect the unit price point and market share. The second driver is the practical aspects of the fabrication process, for example the number and time of injections, which is of great importance for scenarios that rely on injection-based production.

With respect to input costs, the cost of peptone and machinery contribute significantly to the uncertainty but less than public acceptance. CO_2_ taxation was briefly examined. The difference in taxation between BioZEment products and traditional products accounted for less than 1% of the material cost and would therefore have a negligible effect on the current price regimes.

#### Concluding remarks

Despite its high costs, the brick scenario is interesting in an early adaption phase, where one can compete in a niche part of the market with a high price. The main reason for the higher price of BioZEment-based bricks, under the given assumptions, is that the number of casts per time is low compared with traditional brick production, as the injection phase takes time. When limited to the injection process, almost regardless of how short it is possible to make the time between injections and number of injections, BioZEment-based bricks are still likely to be significantly more expensive.

In the precast scenario, the BioZEment-based product can compete on cost and has the possibility to dominate the market if the number of injections can be reduced.

The onsite use of BioZEment has a large potential but relies heavily on the assumption that it is possible to omit the injection process and traditional steel reinforcement. At present, this does not seem very likely, but the potential impact is the largest of the three possible scenarios investigated.

There are possible negative practical aspects related to the BioZEment process and derived products. The LCA showed that ammonia emissions are a potential issue that must be limited as much as possible. This may be particularly challenging for onsite production, where the possibilities for closed-loop systems are limited. As discussed in [4], there also remains a significant amount of work involved to make a strong material that is proven to be durable. To achieve this is one of the main aims of the ongoing research and developmental work.

#### Further work

The public acceptance assumptions are major contributors to the uncertainty. These should be quantified and investigated further in order to balance their contributions in the analysis. This could also aid product development by providing an increased understanding of customer needs. Transparency and the inclusion of consumers and stakeholders in the innovation phase and in the subsequent commercialisation process will be vital to building trust among the consumers.

Regional adaption of the cost and market model should also be performed to prove the scalability assumptions used in the current analysis. It is likely that material costs will scale directly, but there will be a skew in labour versus investment costs to find the local optimum. It is unclear in what direction this will influence the results.

Further scenarios should be investigated to identify possible areas suited for the application of BioZEment-based products. This also includes an exploration of various production methods and layouts that can further reduce cost, production time, and uncertainty.

There are also emerging competitors in the climate-friendly substitute segment. These actors should be investigated and accounted for in the model.

## Supporting information

S1 Appendix Cost Item and Assumption lists (input values to analysis) for brick, prefab and onsite production. (DOCX)
