# Supplementary material for: Towards a low CO2 emission building material employing bacterial metabolism (2/2): Prospects for global warming potential reduction in the concrete industry
Source: PLoS One. 2019 Apr 16;14(4):e0208643. doi: 10.1371/journal.pone.0208643 (PMC6467374; doi:10.1371/journal.pone.0208643)
Supplement: S1 Appendix — (DOCX) [file pone.0208643.s001.docx]

**S1 Appendix**

**Table S1.1: Cost Item list – Brick Production**

| **Description** | **Unit** | ***p10*** | ***p50*** | ***p90*** |
| --- | --- | --- | --- | --- |
|  |  | **Price** | **Price** | **Price** |
| **MATERIAL COSTS:** |  |  |  |  |
| Limestone | NOK/kg | 0.2 | 0.38 | 0.7 |
| Sand | NOK/kg | 0.05 | 0.09 | 0.20 |
| Urea | NOK/kg | 1.5 | 2.1 | 3.0 |
| Glucose | NOK/kg | 2.6 | 3.5 | 4.5 |
| Yeast extract, dry matter | NOK/kg | 700 | 1 400 | 3 000 |
| Peptone | NOK/kg | 1 000 | 2 000 | 5 000 |
| Salt | NOK/kg | 0.200 | 0.336 | 1.000 |
| Bacterial mass, dry matter | NOK/kg | 1 250 | 5 000 | 20 000 |
| Electricity | NOK/kWh | 0.5 | 1.0 | 2.5 |
| Cement | NOK/kg | 1.4 | 1.7 | 2.5 |
| Water | NOK/kg | 0.016 | 0.042 | 0.110 |
| Stone | NOK/kg | 0.1 | 0.2 | 0.38 |
| **LABOUR COSTS:** |  |  |  |  |
| Labor - Base cost | NOK/h | 400 | 500 | 600 |
| Labor - Technician/engineer | NOK/h | 115 % | 120 % | 130 % |
| Labor - Researcher | NOK/h | 125 % | 130 % | 140 % |
| Labor - Admin/sales/log./dist. | NOK/h | 95 % | 100 % | 110 % |
| **FACILITY COSTS:** |  |  |  |  |
| Cost of land | NOK/m^2 | 500 | 1000 | 3000 |
| Local mass handling, site prep | NOK/m^2 | 500 | 1000 | 2000 |
| Prod. facilities construction | NOK/m^2 | 15 000 | 20 000 | 30 000 |
| Insulated Storage facility | NOK/m^2 | 1 500 | 2 000 | 3 000 |
| General maintenance, incl. materials and crew | NOK/m^2/year | 300 | 350 | 600 |
| **EQUIPMENT COSTS:** |  |  |  |  |
| Mixing Plant | NOK/unit | 200 000 | 300 000 | 600 000 |
| Aggregate Batcher | NOK/unit | 200 000 | 250 000 | 500 000 |
| Block Making Machines | NOK/unit | 562 500 | 750 000 | 1 125 000 |
| Pallet Feeders | NOK/unit | 37 500 | 50 000 | 87 500 |
| Truck | NOK/unit | 250 000 | 300 000 | 400 000 |
| Wheel Loader | NOK/unit | 350 000 | 500 000 | 1 000 000 |

**Table S1.2: Assumption list – Brick Production**

| **Description** | **Unit** | ***p10*** | ***p50*** | ***p90*** |
| --- | --- | --- | --- | --- |
|  |  | **Amount** | **Amount** | **Amount** |
| **MATERIAL ASSUMPTIONS:** |  |  |  |  |
| Limestone | kg/ton | 100 | 140 | 200 |
| Urea | kg/ton | 2.8 | 3.4 | 4.2 |
| Glucose | kg/ton | 1.0 | 1.4 | 2.0 |
| Yeast extract, dry matter | kg/ton | 0.030 | 0.045 | 0.070 |
| Peptone | kg/ton | 0.10 | 0.13 | 0.20 |
| Salt | kg/ton | 0.10 | 0.13 | 0.20 |
| Bacterial mass, dry matter | kg/ton | 0.002 | 0.003 | 0.005 |
| Electricity | kWh/ton | 3 | 5 | 10 |
| **TIME ASSUMPTIONS:** |  |  |  |  |
| Time to establish production area | m^2/day | 20 | 25 | 35 |
| Time to establish storage/land area | m^2/day | 40 | 50 | 65 |
| Time for machinery inst. and testing | days | 30 | 50 | 95 |
| Contingency establish factory | days | 5 | 7.5 | 20 |
| **FACILITY ASSUMPTIONS:** |  |  |  |  |
| Production lines | pcs | 2.5 | 3.0 | 3.6 |
| Area of batch and mixing machines | m^2 | 38 | 50 | 75 |
| Area of brick making machines | m^2 | 38 | 50 | 75 |
| Batch/mixing vs brick making ratio trad | - | 0.5 | 1.0 | 2.0 |
| Batch/mixing vs brick making ratio bio | - | 2.0 | 4.0 | 8.0 |
| Additional facilities area | % | 20 % | 30 % | 45 % |
| Land area (addition to facility area) | % | 7.5 % | 10 % | 18 % |
| **PRODUCTION ASSUMPTIONS:** |  |  |  |  |
| Bio. prod. - Production rate | bricks/set | 450 | 750 | 1400 |
| Bio. prod. - Number of injections | - | 5 | 10 | 20 |
| Bio. prod. - Time between injections | h | 1 | 2 | 4 |
| Bio. prod. - Downtime between sets | h | 0.1 | 0.2 | 0.5 |
| Trad. - Production rate | bricks/h | 3 500 | 4 500 | 5 250 |
| Trad. - Start up delay & downtime main. | h/shift | 0.9 | 1 | 1.3 |
| Trad. - Availability (start-up delay and downtime) | % | 78 % | 82 % | 85 % |
| Availability | % | 90 % | 93 % | 94 % |
| Wastage | % | 2 % | 3 % | 5 % |
| **SUPPORT EQUIPMENT ASSUMPTIONS:** |  |  |  |  |
| Truck per bio brick machine | pcs/machine | 0.20 | 0.25 | 0.50 |
| Truck per trad brick machine | pcs/machine | 1.50 | 2.00 | 4.00 |
| Wheel loader per bio mixing plant | pcs/plant | 0.25 | 0.50 | 1.00 |
| Wheel loader per trad mixing plant | pcs/plant | 0.75 | 1.00 | 1.50 |
| **STORAGE ASSUMPTIONS:** |  |  |  |  |
| Storage capacity input | days | 7 | 10 | 14 |
| Storage capacity raw material | ton/m^2 | 2 | 3 | 5 |
| Storage capacity output | days | 14 | 20 | 30 |
| Storage capacity bricks | bricks/m^2 | 750 | 792 | 800 |
| **PERSONNEL ASSUMPTIONS:** |  |  |  |  |
| Bio. Engineer/technician per prod. line | pcs/shift | 1 | 1.5 | 3 |
| Trad. Engineer/technician per prod. line | pcs/shift | 1.25 | 2 | 5 |
| Supervisor/quality control per prod. line | pcs/shift | 0.25 | 0.5 | 1 |
| Logistics per Truck/Wheel loader | pcs/vehicle | 0.75 | 1 | 1.5 |
| **Description** | **Unit** | ***p10*** | ***p50*** | ***p90*** |
|  |  | **Amount** | **Amount** | **Amount** |
| Bio. Admin/Sales/Distribution per prod. line | pcs/day | 0.4 | 0.6 | 1 |
| Trad. Admin/Sales/Distribution per prod. line | pcs/day | 2 | 3.5 | 7 |
| Trad. Supervisor/quality control per prod. line | pcs/shift | 0.75 | 1 | 1.75 |
| **MAINTENANCE ASSUMPTIONS:** |  |  |  |  |
| Maintenance factor - low wear and tear | %/y of cost price | 2 % | 4 % | 10 % |
| Maintenance factor - high wear and tear | %/y of cost price | 5 % | 10 % | 25 % |
| Main. complexity factor - Bio vs. Trad. | % | 100 % | 150 % | 250 % |
| **MARKET ASSUMPTIONS:** |  |  |  |  |
| Size of market for concrete | megaton | 20 160 | 22 400 | 24 640 |
| Share of brick concrete | % | 0.15 | 0.2 | 0.24 |
| Profit margins | % | 0.10 | 0.15 | 0.25 |
| Fully dev. comp. | - | 2 | 3 | 5 |
| **GWP ASSUMPTIONS:** |  |  |  |  |
| GWP BioZEment | kg/ton bioZEment | 12.5 | 17.0 | 22.6 |
| GWP traditional concrete | kg/ton concrete | 70.0 | 75.6 | 115.6 |
| **PUBLIC ACCEPTANCE ASSUMPTIONS:** |  |  |  |  |
| Desire for new product | % | 75 % | 83 % | 90 % |
| Fear of contamination | % | 105 % | 110 % | 115 % |
| **GENERAL ASSUMPTIONS:** |  |  |  |  |
| Project write off time | years | 7 | 10 | 17 |

**Table S1.3: Cost Item list – Prefab Production**

| **Description** | **Unit** | ***p10*** | ***p50*** | ***p90*** |
| --- | --- | --- | --- | --- |
|  |  | **Price** | **Price** | **Price** |
| **MATERIAL COSTS:** |  |  |  |  |
| Limestone | NOK/kg | 0.2 | 0.38 | 0.7 |
| Sand | NOK/kg | 0.05 | 0.09 | 0.2 |
| Urea | NOK/kg | 1.5 | 2.1 | 3 |
| Glucose | NOK/kg | 2.55 | 3.5 | 4.5 |
| Yeast extract, dry matter | NOK/kg | 700 | 1 400 | 3 000 |
| Peptone | NOK/kg | 1 000 | 2 000 | 5 000 |
| Salt | NOK/kg | 0.2 | 0.336 | 1 |
| Bacterial mass, dry matter | NOK/kg | 1 250 | 5 000 | 20 000 |
| Electricity | NOK/kWh | 0.5 | 1 | 2.5 |
| Strand in coil | NOK/kg | 4 | 6 | 10 |
| Oil (prior to cast) | NOK/l | 400 | 500 | 800 |
| Cleaner Fluid (prior to cast) | NOK/l | 10 | 30 | 100 |
| Cement | NOK/kg | 1.4 | 1.7 | 2.5 |
| Water | NOK/kg | 0.016 | 0.042 | 0.11 |
| Stone | NOK/kg | 0.1 | 0.2 | 0.38 |
| **LABOUR COSTS:** |  |  |  |  |
| Labor - Base cost | NOK/h | 450 | 500 | 550 |
| Labor - Technician/engineer | % | 115 % | 120 % | 130 % |
| Labor - Researcher | % | 125 % | 130 % | 140 % |
| Labor - Logistics/distribution | % | 95 % | 100 % | 105 % |
| Labor - Admin | % | 95 % | 100 % | 110 % |
| **FACILITY COSTS:** |  |  |  |  |
| **Description** | **Unit** | ***p10*** | ***p50*** | ***p90*** |
|  |  | **Price** | **Price** | **Price** |
| Cost of land | NOK/m^2^ | 500 | 1000 | 3000 |
| Local mass handling, site prep | NOK/m^2^ | 500 | 1000 | 2000 |
| Prod. facilities construction | NOK/m^2^ | 15 000 | 20 000 | 30 000 |
| Insulated Storage facility | NOK/m^2^ | 1 500 | 2000 | 3 000 |
| General maintenance, incl. materials and crew | NOK/m2/year | 750 | 1000 | 1250 |
| Equipment maintenance, incl. materials and crew | NOK/h | 1 250 | 1 500 | 2 000 |
| **EQUIPMENT COSTS:** |  |  |  |  |
| Batching and Mixing Plant | NOK/unit | 11 250 000 | 15 000 000 | 22 500 000 |
| Prod. Equipment - Beds | NOK/m | 5 625 | 7 500 | 13 125 |
| Prod. Equipment - Preparers | NOK/unit | 3 750 000 | 5 000 000 | 8 750 000 |
| Prod. Equipment - Shuttles | NOK/unit | 5 250 000 | 7 000 000 | 12 250 000 |
| Prod. Equipment - Distributors | NOK/unit | 750 000 | 1 000 000 | 1 750 000 |
| Prod. Equipment - Prestressing Units | NOK/unit | 2 250 000 | 3 000 000 | 5 250 000 |
| Prod. Equipment - Extruders | NOK/unit | 5 250 000 | 7 000 000 | 12 250 000 |
| Prod. Equipment - Modifiers | NOK/unit | 2 250 000 | 3 000 000 | 5 250 000 |
| Prod. Equipment - Plotters | NOK/unit | 1 500 000 | 2 000 000 | 3 500 000 |
| Prod. Equipment - Maturity Controllers | NOK/unit | 1 500 000 | 2 000 000 | 3 500 000 |
| Prod. Equipment - Loopers | NOK/unit | 750 000 | 1 000 000 | 1 750 000 |
| Prod. Equipment - Saws | NOK/unit | 3 000 000 | 4 000 000 | 7 000 000 |
| Prod. Equipment - Lifting Beams | NOK/unit | 112 500 | 150 000 | 262 500 |
| Prod. Equipment - Water Hole Drillers | NOK/unit | 750 000 | 1 000 000 | 1 750 000 |
| Prod. Equipment - Wagons | NOK/unit | 750 000 | 1 000 000 | 1 750 000 |
| Prod. Equipment - Storage Clamps | NOK/unit | 225 000 | 300 000 | 525 000 |
| Prod. Equipment - Computer system | NOK/unit | 3 750 000 | 5 000 000 | 8 750 000 |

**Table S1.4: Assumption list – Prefab Production**

| **Description** | **Unit** | ***p10*** | ***p50*** | ***p90*** |
| --- | --- | --- | --- | --- |
|  |  | **Amount** | **Amount** | **Amount** |
| **MATERIAL ASSUMPTIONS:** |  |  |  |  |
| Limestone | kg/ton | 100 | 140 | 200 |
| Urea | kg/ton | 2.8 | 3.4 | 4.2 |
| Glucose | kg/ton | 1 | 1.4 | 2 |
| Yeast extract, dry matter | kg/ton | 0.03 | 0.045 | 0.07 |
| Peptone | kg/ton | 0.1 | 0.13 | 0.2 |
| Salt | kg/ton | 0.1 | 0.13 | 0.2 |
| Bacterial mass, dry matter | kg/ton | 0.002 | 0.003 | 0.005 |
| Electricity | kWh/ton | 3 | 5 | 10 |
| Oil | l/ton | 0.03 | 0.05 | 0.1 |
| Cleaner Fluid | l/ton | 0.005 | 0.01 | 0.02 |
| **TIME ASSUMPTIONS:** |  |  |  |  |
| Time to establish production area | m2/day | 20 | 25 | 35 |
| Time to establish storage/land area | m2/day | 40 | 50 | 65 |
| Time for machinery installation and testing | days | 30 | 50 | 95 |
| Contingency establish factory | days | 5 | 7.5 | 20 |
| **PRODUCTION ASSUMPTIONS:** |  |  |  |  |
| Availability | % | 90 % | 95 % | 98 % |
| Wastage | % | 1 % | 3 % | 9 % |
| **Description** | **Unit** | ***p10*** | ***p50*** | ***p90*** |
|  |  | **Amount** | **Amount** | **Amount** |
| Land area | m2 | 8 000 | 9 000 | 10 000 |
| Size of facility | m2 | 4 500 | 5 000 | 6 000 |
| No. of cast per day trad | cast/day | 1.20 | 1.30 | 1.60 |
| Number of injections | - | 5 | 10 | 20 |
| Time between injections | h | 1.1 | 1.6 | 3.3 |
| Reset time between injections | h | 1.1 | 1.25 | 1.7 |
| **PERSONNEL ASSUMPTIONS:** |  |  |  |  |
| Personnel - Engineer/technician | pcs/shift | 6 | 7 | 9 |
| Personnel - Supervisor/quality control | pcs/shift | 1 | 1.5 | 4 |
| Personnel - Logistics | pcs/shift | 2 | 3 | 4.5 |
| Personnel - Admin | pcs/shift | 2 | 3 | 4.5 |
| Personnel - Sales | pcs/shift | 1 | 1.5 | 3 |
| Personnel - Distribution | pcs/shift | 1 | 1.5 | 3 |
| **MAINTENANCE ASSUMPTIONS:** |  |  |  |  |
| Maintenance factor - low wear and tear | %/y of cost price | 2 % | 4 % | 10 % |
| Maintenance factor - medium wear and tear | %/y of cost price | 7 % | 10 % | 17 % |
| Maintenance factor - high wear and tear | %/y of cost price | 5 % | 10 % | 25 % |
| Maintenance factor - very high wear and tear | %/y of cost price | 10 % | 15 % | 25 % |
| **GENERAL ASSUMPTIONS:** |  |  |  |  |
| Project write off time | years | 7 | 10 | 17 |
| **MARKET ASSUMPTIONS:** |  |  |  |  |
| Size of market for concrete | megaton | 20 160 | 22 400 | 24 640 |
| Share of prefab concrete | % | 0.18 | 0.25 | 0.29 |
| Profit margins | % | 0.1 | 0.15 | 0.25 |
| Fully dev. comp. | - | 2 | 3 | 5 |
| **PUBLIC ACCEPTANCE ASSUMPTIONS:** |  |  |  |  |
| Desire for new product | % | 75 % | 83 % | 90 % |
| Fear of contamination | % | 105 % | 110 % | 115 % |
| **GWP ASSUMPTIONS:** |  |  |  |  |
| GWP BioZEment | kg/ton bioZEment | 12.5 | 17.0 | 22.6 |
| GWP traditional concrete | kg/ton concrete | 70.00 | 75.60 | 115.60 |

**Table S1.5: Cost Item list – Onsite Production**

| **Description** | **Unit** | ***p10*** | ***p50*** | ***p90*** |
| --- | --- | --- | --- | --- |
|  |  | **Price** | **Price** | **Price** |
| **MATERIAL COSTS:** |  |  |  |  |
| Limestone | NOK/kg | 0.2 | 0.38 | 0.7 |
| Sand | NOK/kg | 0.05 | 0.09 | 0.2 |
| Urea | NOK/kg | 1.5 | 2.1 | 3 |
| Glucose | NOK/kg | 3 | 4.8 | 20 |
| Yeast extract (in bacteria medium) | NOK/kg | 700 | 1400 | 3000 |
| Peptone (in bacteria medium) | NOK/kg | 1 000 | 2 000 | 5 000 |
| Salt (in bacteria medium) | NOK/kg | 0.2 | 0.336 | 1 |
| Bacterial mass, spores | NOK/kg | 1 250 | 5 000 | 20 000 |
| Electricity | NOK/kWh | 0.7 | 1 | 1.5 |
| Reinforcement | NOK/kg | 7 | 9 | 12 |
| Scaffolding, wall | NOK/m^2 | 600 | 700 | 900 |
| Cement | NOK/kg | 1.4 | 1.7 | 2.5 |
| Water | NOK/kg | 0.016 | 0.042 | 0.11 |
| Stone | NOK/kg | 0.1 | 0.2 | 0.38 |
| **PRODUCTION COSTS:** |  |  |  |  |
| Labor - Base cost | NOK/h | 450 | 500 | 550 |
| Labor - Admin | % | 95 % | 100 % | 110 % |
| Labor - Manual labor | % | 110 % | 140 % | 190 % |
| Scaffolding, wall | NOK/h | 300 | 350 | 400 |
| Concrete transport | NOK/m^3 | 150 | 200 | 300 |
| **FACILITY COSTS:** |  |  |  |  |
| Cost of mobile mixing plant | NOK/m^3 | 1805.25 | 2407 | 3129.1 |
| Work mixing plant | NOK/m^3 | 81 | 108 | 140.4 |
| ROI mixing concrete mix | NOK/m^3 | 200 | 0 | 400 |

**Table S6: Assumption list – Onsite Production**

| **Description** | **Unit** | ***p10*** | ***p50*** | ***p90*** |
| --- | --- | --- | --- | --- |
|  |  | **Amount** | **Amount** | **Amount** |
| **MATERIAL ASSUMPTIONS:** |  |  |  |  |
| Limestone | kg/ton | 100 | 140 | 200 |
| Urea | kg/ton | 2.8 | 3.4 | 4.2 |
| Glucose | kg/ton | 1 | 1.4 | 2 |
| Yeast extract (in bacteria medium) | kg/ton | 0.03 | 0.045 | 0.07 |
| Peptone (in bacteria medium) | kg/ton | 0.1 | 0.13 | 0.2 |
| Salt (in bacteria medium) | kg/ton | 0.1 | 0.13 | 0.2 |
| Bacterial mass, spores | kg/ton | 0.002 | 0.003 | 0.005 |
| Electricity | kWh/ton | 3 | 5 | 10 |
| **PROJECT ASSUMPTIONS:** |  |  |  |  |
| Time to establish factory site | m^2/day | 30 | 40 | 60 |
| Construction speed | m^2/day | 3 | 5 | 10 |
| Profit margins | % | 0.1 | 0.15 | 0.23 |
| Work complexity factor | % | 0.1 | 0.2 | 0.5 |
| **PRODUCTION ASSUMPTIONS:** |  |  |  |  |
| Bacteria mass multiplier | - | 5.00 | 10.00 | 20.00 |
| Time scaffolding. Inc tear down | h/m^2 | 0.70 | 1.00 | 2.00 |
| Time. Rebar | h/m^3 | 2 | 3 | 5 |
| Time. Fresh concrete work | h/m^3 | 0.4 | 0.6 | 1 |
| Worker efficiency | % | 0.6 | 0.7 | 0.9 |
| Time. Admin and sales | h/m^3 | 0.02 | 0.03 | 0.07 |
| **MARKET ASSUMPTIONS:** |  |  |  |  |
| Size of market for concrete | megaton | 20 160 | 22 400 | 24 640 |
| Share of on-site concrete | % | 0.18 | 0.25 | 0.29 |
| Fully dev. comp. | - | 2 | 3 | 5 |
| **GWP ASSUMPTIONS:** |  |  |  |  |
| GWP BioZEment | kg/ton bioZEment | 12.5 | 17.0 | 22.6 |
| GWP traditional concrete | kg/ton concrete | 70.0 | 75.6 | 115.6 |
| **PUBLIC ACCEPTANCE ASSUMPTIONS:** |  |  |  |  |
| Desire for new product | % | 0.75 | 0.825 | 0.9 |
| Fear of contamination | % | 1.05 | 1.1 | 1.15 |
